# Distribution and impact of p16^INK4A+^ senescent cells in elderly tissues: a focus on senescent immune cell and epithelial dysfunction

**DOI:** 10.1038/s12276-024-01354-4

**Published:** 2024-12-02

**Authors:** Soon Sang Park, Young-Kyoung Lee, Young Hwa Kim, So Hyun Park, Hee Young Kang, Jin Cheol Kim, Dong Jun Kim, Su Bin Lim, Gyesoon Yoon, Jang-Hee Kim, Yong Won Choi, Tae Jun Park

**Affiliations:** 1 Inflammaging Translational Research Center, Ajou University Medical Center, Suwon, Korea; 2 Department of Biochemistry and Molecular Biology, Ajou University School of Medicine, Suwon, Korea; 3 Department of Pathology, Ajou University School of Medicine, Suwon, Korea; 4 Department of Dermatology, Ajou University School of Medicine, Suwon, Korea; 5 Department of Biomedical Sciences, Ajou University Graduate School of Medicine, Suwon, Korea; 6 Department of Hematology and Oncology, Ajou University School of Medicine, Suwon, Korea

**Keywords:** Translational research, Cellular signalling networks

## Abstract

Cellular senescence, recognized as a key hallmark of aging, leads to the accumulation of senescent cells in various tissues over time. While the detrimental effects of these cells on age-related pathological conditions are well-documented, there is still limited information about how senescent cells are distributed in normal tissues of both young and aged organs. Our research indicates that fully senescent p16^INK4A+^ cells are rarely identified in the parenchyma of organic tissues and in the stromal cells crucial for structural maintenance, such as fibroblasts and smooth muscle cells. Instead, p16^INK4A+^ cells are more commonly found in immune cells, whether they reside in the organ or are infiltrating. Notably, p16^INK4A+^ senescent T cells have been observed to induce apoptosis and inflammation in colonic epithelial cells through Granzyme A-PARs signaling, compromising the integrity of the epithelial lining. This study showed that the senescence of immune cells could affect the phenotypical change of the parenchymal cells in the elderly and suggests that targeting immunosenescence might be a strategy to control functional decline in this population.

## Immune cell senescence impacts aging tissue integrity

Cellular senescence accumulates in tissues as we age, causing dysfunction and inflammation. However, the specific cell types that become senescent and their removal are not well understood. Researchers aimed to map senescent cells in young and elderly tissues, creating a “Senescence Atlas.” This study involved analyzing human tissues and mouse tissues using immunohistochemistry and single-cell RNA-sequencing. They focused on p16INK4A and found that senescent cells were rare in both young and elderly tissues. However, immune cells, especially T cells, showed increased senescence in elderly tissues. Results showed that senescent T cells release granzyme A, which activates protease-activated receptors in epithelial cells, leading to inflammation and cell death. This suggests that targeting senescent immune cells could improve organ function in the elderly. This summary was initially drafted using artificial intelligence, then revised and fact-checked by the author.

## Introduction

Cellular senescence, as a major player among hallmarks of aging^1^, has been reported as being able to accumulate senescent cells in various tissues during aging process^2–5^. Cellular senescence can cause a halt in the proliferation of functional cells, ultimately resulting in organic dysfunction^6–9^ and induce sterile chronic inflammation through the secretion of senescence-associated secretory phenotypes (SASPs), which are known as ‘inflammaging‘^10,11^. Previous studies applying senolytics^12^ or selective cytotoxicity in p16^INK4A^-overexpressed cells in aged mice^6^ have been supported the notion that removal of senescent cells can be alleviate not all but many aging-related phenotypes and lead to the prolongation of life span. Although the final phenotypes resulting from the removal of senescent cells have been confirmed in multiple previous studies^3,6,13^, information about the specific cell types that accumulate as senescent cells and their removal remains scarce.

Organs are composed of two major components: the parenchyma and the stroma. Parenchymal cells, responsible for executing organ-specific functions, often exhibit rapid proliferation and turnover rates due to their frequent exposure to external stimuli^14,15^. Examples include gastrointestinal tract epithelial cells and skin keratinocytes. Conversely, the tissue stroma can be further categorized into cells providing structural support and immune cells^16^. Cells providing structural support (hereinafter referred as structural stromal cells), such as fibroblasts and smooth muscle cells produce extracellular matrix (ECM) components and maintain tissue structures^17^. These cells exhibit slower self-renewal largely because they are shielded by parenchymal cells and are less exposed to external stress^18,19^. The remaining stromal cells are immune cells, which may be resident, such as liver Kupffer cells and skin Langerhans cells, or infiltrating, such as bone marrow-derived cells and lymphocytes. These cells are involved in protecting organs from foreign invaders, chronic inflammation, and tissue regeneration related to the aging process^20^. Although the activation mechanisms vary by immune cell type, most immune cells in normal conditions remain in the quiescent state in blood or their resident organs until signs of infection or inflammation trigger activation^21^. Therefore, each organ is composed of various cell types with differing proliferation capacities, making it crucial to understand which cells undergo senescence in elderly individuals. Given the different proliferation capacities and turnover rates of each cell type or cellular origin^19^, we hypothesized that the degree of senescence progression may vary among these cell types in the tissues of the elderly. Understanding this variation is crucial for comprehending the overall process of tissue and organ aging, as well as for developing future anti-aging interventions. Therefore, in this study, we aim to provide information about the proportion of senescent cells in young and elderly individuals and how they are distributed in the tissue parenchyma and stroma, respectively. We refer to this as the “Senescence Atlas.” The “Senescence Atlas” would help link the concepts of individual aging and cellular senescence. Additionally, we aimed to understand how accumulated senescent cells in the stroma affect tissue parenchymal function.

## Materials and methods

### Human sample preparation

This study adheres to the Declaration of Helsinki and received approval from the Institutional Review Board of Ajou University Hospital (AJIRB-BMR-OBS-20-552, Suwon, Korea), with informed consent obtained from participants. Various normal tissues (colon, small intestine, lung, liver, thyroid, skin, and spleen) were collected from surgically resected specimens adjacent to pathological areas, with the guidance of an experienced pathologist. Normal tissues were sourced from histologically non-pathological regions around the resection margins, located 5–12 cm away from the pathological lesions. These tissues were individually sampled from representative areas by an experienced pathologist, immediately fixed, and processed into formalin-fixed paraffin-embedded (FFPE) blocks, following the tissue specimen regulations of Ajou University Hospital. The patient information used in the study can be found in Supplementary Table 1–7. To avoid identification of individual patients and ethical issues, we provide summary statistics of the patient information according to the organ studied.

### Animal experiments

Male and female C57BL/6 J mice, aged 4 months and 12 months, were purchased from the Korea Basic Science Institute (KBSI) in Gwangju, Korea. The 12-month-old mice were maintained in a clean room system until they reached 18 and 22 months of age. Mice from each age group were sacrificed at 4 months, 18 months, and 22 months old. All animal procedures were approved by the Institutional Animal Research Ethics Committee at Ajou University Medical Center (approval number: 2020-0051).

### Immunohistochemistry (IHC) and immunofluorescence (IF)

IHC staining of FFPE tissue Section (4-μm) was performed on Benchmark XT automated IHC stainer (Ventana Medical Systems Inc., Tucson, AZ). The primary antibodies used for human samples were as follows: p16^INK4A^, predilution (805–4713, Roche, Basal, Switzerland); human Ki67, 1:3,000 (M7240, Dako, Glostrup, Denmark); H3K9me3, 1:500 (ab176916, Abcam, Cambridge, MA); CD3, 1:150 (ab135372, Abcam); CD19, 1:100 (ab227688, Abcam), CD68, 1:2,500 (NBP2-48923, Novus Biologicals, Centennial, CO); PAR1, 1:200 (ab233741, Abcam); PAR2, 1:100 (ab184673, Abcam); Granzyme A, 1:100 (ab209205, Abcam); Cleaved caspase-3, 1:400 (9661S, Cell Signaling Technology, Danvers, MA); IL8, 1:300 (27095-1-AP, Proteintech, Rosemont, IL) IFNα 1:200 (PA5-119649, Thermo Fisher Scientific, Waltham, MA); IFNβ 1:500 (PA5-102429, Thermo Fisher Scientific). The primary antibodies used for mouse samples were as follows: p16^INK4A^, 1:300. (ab189034, Abcam); mouse Ki67, 1:200 (ab16667, Abcam); IHC image acquisition was conducted using a Aperio ScanScope CS scanning microscope (Aperio Technologies Inc., Vista, CA). For IF staining of FFPE tissue sections, the following primary antibodies were incubated overnight at 4 °C: p16^INK4A^, predilution (805–4713, Roche); p21^Waf1^, 1:100 (ab109520, Abcam). Slides were washed twice with phosphate buffered saline and incubated with appropriately conjugated secondary antibodies for 1 h at room temperature. Secondary antibodies used were as follows: Alexa Fluor 488, 1:300 (ab150113, Abcam); Alexa Fluor 594, 1:300 (ab150080, Abcam). The stained cells were visualized with a LSM 710 confocal laser microscope (Zeiss, Oberkochen, Germany). Multiplex IHC staining was performed using BOND Polymer Refine Detection kit (DS9800, Leica Biosystems, Wetzlar, Germany), BOND Polymer Refine Red Detection kit (DS9390, Leica Biosystems), and BOND Green Chromogen kit (DC9913, Leica Biosystems) in the BOND-III fully automated IHC staining system (22.2201, Leica Biosystems).

### SA-β-Gal Staining

The 8 μm-sliced frozen tissue slides were fixed with 10% formalin (HT501128, Sigma-Aldrich, Saint Louis, MO) for 1 min. Subsequently, the slide was incubated with SA-β-Gal working solution (1 mg/ml X-gal; 40 mM citric acid pH 5.8; 5 mM potassium ferrocyanide; 5 mM; potassium ferricyanide; 150 mM NaCl; 2 mM MgCl_2_) for 14 h at 37 °C. The stained images were visualized with a Aperio ScanScope CS scanning microscope (Aperio Technologies Inc.).

### Cell culture

Human primary colonic epithelial cells (HCoEpiC) were purchased from Cell Biologics, Inc. (Chicago, IL) and cultured in Human Epithelial Cell Basal Medium (Cell Biologics, Inc.) at 37 °C in a humidified incubator with 5% CO_2_. Fresh blood samples were collected from the Korean Red Cross Blood Services with the approval of the Institutional Review Board of Ajou University Hospital (AJIRB-BMR-EXP-22-246). Peripheral blood mononuclear cells (PBMCs) were obtained by density gradient centrifugation with Ficoll-Paque Plus (17144002, Cytiva, Marlborough, MA). T cells were isolated from PBMCs using the Naive Pan T Cell Isolation Kit (130-097-095, Miltenyi Biotec, Auburn, CA) according to the manufacturer’s instructions. Cells were cultured at 1 × 10^6^ in AIM-V complete medium (12055091, Thermo Fisher Scientific) supplemented with 50 IU/ml recombinant human IL-2 (rhIL-2) (11011456001, Roche) and 5% heat-inactivated human AB serum (H4522, Sigma-Aldrich) in a 24-well culture plate at 37 °C in a humidified incubator with 5% CO_2_. T cell stimulation was performed using Dynabeads Human T-Activator CD3/CD28 (11161D, Thermo Fisher Scientific) for 3 days. After the removal of the anti-CD3/28 microbead cocktail, the total cell number and the population doubling level were measured every 2–4 days. Cells were collected along with the conditioned medium (CM). 1 × 10^6^ T cells were seeded when they were subcultured. The CM was harvested from the culture medium of young or senescent T cells (3 × 10^6^ cells) and filtered with 0.2 μm syringe filter. The cumulative population doubling levels were calculated as follows:

$$\,\begin{array}{c}{\rm{cPDL}}={\rm{cPD}}{{\rm{L}}}_{0}+3.322\,\times \log \left(\frac{{C}_{f}}{{C}_{i}}\right)\\ ({{\rm{PDL}}}_{0}:{\rm{previous\; cPDL}},Ci:{\rm{initial\; cell\; number}},\,Cf:{\rm{final\; cell\; number}})\end{array}$$

### C_12_FDG staining

To analyze SA-β-gal activity, ImaGene Green^TM^ C_12_FDG lacZ gene Expression Kit (I2904, Invitrogen, Carlsbad, CA) was used according to the manufacturer’s instructions. Briefly, young or senescent T cells was pre-incubated with 300 μM chloroquine for 30 min at 37 °C and incubated with 33 μM substrate reagent for 1 h at 37 °C. To exclude dead cells, cells were stained with Zombie-NIR (77184, Biolegend, San Diego, CA) dye. Stained cells were washed and resuspended in PBS, and analyzed by flow cytometry (FACSAria III, Becton Dickinson, Bedford, MA). Mean values of arbitrary fluorescence unit of 10,000 live cells (Zombie-NIR negative cells) were obtained.

### ELISA analysis

Human Granzyme A ELISA Kit (ELH-GZMA-1, RayBiotech, Peachtree Corners, GA) was used for quantitative measurement of granzyme A concentration in collected CM according to the manufacturer’s instructions and measurement on spectrophotometer (Eon microplate reader, BioTek Instruments, Inc.).

### Coculture and CM treatment of colonic epithelial cells with T cells

For co-culture, human colon epithelial cells were seeded in a 24-well plate (2 × 10^4^ cells /well). After 1 days, human colon epithelial cells co-cultured with young or senescent T cells, which were seeded onto the upper chamber of the SPLInsert™ (0.4-µm pore size, 24 well, SPL Life Sciences Co., Ltd, Pocheon, Korea). For CM treatment experiments, human colon epithelial cells were seeded onto 24-well plates (2 × 10^4^ cells/well) and allowed to adhere for 1day. Culture medium was then replaced with mixed culture medium (culture medium:CM = 4:1).

### Cleaved caspase 3/7 activity measurement

Caspase 3/7 activity was quantified using the Caspase-Glo® 3/7 Assay System (G8090, Promega, Mannheim, Germany) according to the manufacturer’s instructions and measurement on a luminometer (Synergy 2 Multi-Mode Reader, BioTek Instruments, Inc., Winooski, VT).

### Immunocytochemistry (ICC)

Cells were fixed with 4% paraformaldehyde and permeabilized with 0.1% Triton X-100 and blocked with 1% BSA solution. Primary cleaved caspase 3 antibody (1:400, 9661S, Cell Signaling Technology) and Alexa Fluor 488-conjugated anti-rabbit secondary antibody (1:300, ab150113, Abcam) were used to detect cleaved caspase 3. Nuclei were counter-stained with DAPI (1:10,000, D3571, Invitrogen) and the stained cells were visualized with a Lionheart FX automated microscope (BioTek Instruments, Winooski, VT). The percentage of cleaved caspase 3 positive cells were counted by the ImageJ software (NIH, Bethesda, MD, freeware imaging software).

### PARs agonist experiments

Human colon epithelial cells were pre-treated with PAR1 antagonist (100 nM, SCH79797, HY-14993, MedChemExpress, Monmouth Junction, NJ) or PAR2 antagonist (20 μM, AZ3451, HY-112558, MedChemExpress) for 1 h and incubated with recombinant human granzyme A protein (50 ng/ml, ab157288, Abcam) for 1day.

### Granzyme A inhibitor treatment

Nafamostat mesylate (NM, HY-B0190A, MedChemExpress) was incubated with CM from senescent T cells (culture medium: CM = 4:1) or recombinant human granzyme A protein (50 ng/ml) for 1 h at room temperature. These mixtures were treated to human colonic epithelial cells (HCoEpiC, Cell Biologics, Inc.) for 1 day.

### Real-time PCR

RNA was extracted using NucleoSpin RNA Plus extraction kit (740984, MACHEREY-NAGEL, Düren, Germany). First-strand cDNA was produced from 100 ng total RNA using the SuperScript^TM^ IV cDNA Synthesis Kit (18091050, Thermo Fisher Scientific). Real-time PCR was conducted with iQ^TM^ SYBR^®^ Green Supermix (1708880, Bio-Rad, Hercules, CA) using the following conditions: Initial activation at 95 °C 3 min, by 50 cycles of 95 °C for 10 sec and 60 °C for 30 sec. Used primers (Cosmogenetech co, Ltd, Seoul, Korea) are as follows: p16^INK4A^ (*CDKN2A*), 5*'*-CTC GTG CTG ATG CTA CTG AGG A-3*'*, 5*'*-GGT CGG CGC AGT TGG GCT CC-3*'*; p21^Waf1^ (*CDKN1A*), 5*'*-ATT AGC AGC GGA ACA AGG AGT CAG ACA T-3*'*, 5*'*-CTG TGA AAG ACA CAG AAC AGT ACA GGG T-3*'*; *CD28*, 5*'*-GAG AAG AGC AAT GGA ACC ATT ATC-3*'*, 5*'*-TAG CAA GCC AGG ACT CCA CCA A-3*'*; CD57 (*B3GAT1*), 5*'*-GAA AGC AGC CTC CTT CGA GAA C-3*'*, 5*'*-CCT CAT TCA CCA GCA CTG GCT T-3*'*; Granzyme A (*GZMA*), 5*'*- CCA CAC GCG AAG GTG ACC TTA A-3*'*, 5*'*- CCT GCA ACT TGG CAC ATG GTT C-3*'*; IL-8, 5*'*- ATG ACT TCC AAG CTG GCC GTG GCT-3*'*, 5*'*- TCT CAG CCC TCT TCA AAA ACT TCT-3*'*; IL-6, 5*'*-AAG CCA GAG CTG TGC AGA TGA GTA-3*'*, 5*'*-TGT CCT GCA GCC ACT GGT TC-3*'*; IL1β, 5*'*-CTG TCC TGC GTG TTG AAA GA-3*'*, 5*'*-TTG GGT AAT TTT TGG GAT CTA CA-3*'*; CXCL1, 5*'*-AGC TTG CCT CAA TCC TGC AT-3*'*, 5*'*-CCT CTG CAG CTG TGT CTC TC-3*'*; CCL2, 5*'*-AGC AGC AAG TGT CCC AAA GA-3*'*,5'-TTG GGT TTG CTT GTC CAG GT-3*'*; TNF-α, 5*'*-CTG TCC TGC GTG TTG AAA GA-3*'*, 5*'*-TTG GGT AAT TTT TGG GAT CTA CA-3*'*; Granzyme B (*GZMB*), 5*'*- CGA CAG TAC CAT TGA GTT GTG CG-3*'*, 5*'*- TTC GTC CAT AGG AGA CAA TGC CC-3’; Proteinase 3, 5*'*- ACG ACG TTC TCC TCA TCC AG-3’, 5*'*- GTG ACC ACG GTG ACA TTG AG-3*'*; Cathepsin G, 5*'*- ACA CCC AGC AAC ACA TCA CTG C-3*'*, 5*'*- GGT TCA CGT TTC GAT TCC GTC TG-3*'*; β-actin, 5*'*- CCC TGG CAC CCA GCA C-3*'*, 5*'*-GCC GAT CCA CAC GGA GTA C-3’. Thrombin, 5*'*- GAG CCA ACA CCT TCT TGG AG-3*'*, 5*'*- GAG CCA ACA CCT TCT TGG AG-3*'*; Plasmin, 5*'*- GTT TGG GAA TGG GAA AGG AT-3*'*, 5*'*- TAG CAC CAG GGA CCA CCT AC-3*'*; MMP1, 5*'*- AAG CGT GTG ACA GTA AGC TA-3*'*, 5*'*- AAC CGG ACT TCA TCT CTG -3*'*; MMP2, 5*'*- GTG CTG AAG GAC ACA CTA AAG AAG A-3’, 5*'*- TTG CCA TCC TTC TCA AAG TTG TAG G-3*'*; IFNα, 5*'*- AGA AGG CTC CAG CCA TCT CTG T -3*'*, 5*'*- TGC TGG TAG AGT TCG GTG CAG A -3*'*; IFNβ, 5*'*- CTT GGA TTC CTA CAA AGA AGC AGC -3*'*, 5*'*- TCC TCC TTC TGG AAC TGC TGC A -3*'*.

### scRNA-sequencing analysis for human tissues

In the analysis of cells from normal tissue, the publicly available scRNA-seq datasets, GSE178341 (peri-lesional normal colon)^22^, GSE130973 (peri-lesional normal skin)^23^, SYN21041850 (peri-lesional normal lung)^24^, and GSE149614 (peri-lesional normal liver)^25^ were utilized. The offered count data and metadata were loaded into “Seurat Object” using the R (version 4.0.3) “Seurat” package^26,27^ (version 4.3.0) except the skin sample. GSE130973 (normal skin)^25^ offered a processed “rds” file for processed Seurat object, and the preprocessed data are used for further analysis. Following normalization was performed using the “NormalizeData” function, and principal component analysis (PCA) was performed using the “RunPCA” function. To determine dimensionality, “JackStraw” function was performed with 100 times of replications. Detailed patient information, coefficients used for preprocess, and cell annotation markers in each dataset can be found in Supplementary Data.

### Cell-cell interaction analysis

An rds file from peri-lesional normal colonic tissue from GSE178341^22^ preprocessed above section was utilized for cell-cell interaction analysis. T cells, annotated above, are further subdivided according to the p16^INK4A+^ (*CDKN2A*) expression: *CDKN2A*^-^ and *CDKN2A*^+^. Finally, eight clusters of cells (plasma cells, fibroblasts, bone marrow cells, p16^INK4A+^ T cells, p16^INK4A-^ mast cells, epithelial cells, and endothelial/smooth muscle cells) were analyzed. A CellChat library (v 1.6.1) was used for this analysis^28^. A CellChat object was created using the “create CellChat” function. The “computeCommunProb”, “filterCommunication”, “computeCommunProbPathway”, and “aggregateNet” functions were sequentially performed to project the preprocessed data. The graphs were visualized using the “netVisual_circle”, “netAnalysis_signalingRole_scatter”, and “netAnalysis_signalingRole_heatmap” functions. Specific pathways were further analyzed using the code “pathways.show <- c(“specific pathway”)”.

### scRNA-sequencing analysis for mouse tissues

A public scRNA-seq dataset of mouse tissue, GSE109774 (Tabula Muris)^29^, was utilized for a comprehensive analysis. The publicly available Python-based FACS-sorted Anndata, provided by The Tabula Muris Consortium, includes tissue samples collected from 18 C57BL/6JN mice at different ages, specifically 3 months (*n* = 10), 18 months (*n* = 4), and 24 months (*n* = 4). The tissues represented in the dataset are lung, liver, colon, skin, and others. To begin the analysis, the annotation data (h5ad files), which includes gene count tables and associated metadata variables, were loaded into the Python environment using the Scanpy package (v. 1.9.3)^30^. The visualization of gene expression patterns such as “*Cdkn2a*” was conducted based on different factors such as tissues, cell types, and age groups using UMAP projection (scanpy.pl.umap) and a dotplot (scanpy.pl.dotplot).

## Results

### Distribution of p16^INK4A+^ senescent cells in young and aged normal tissues

**Fig. 1:**
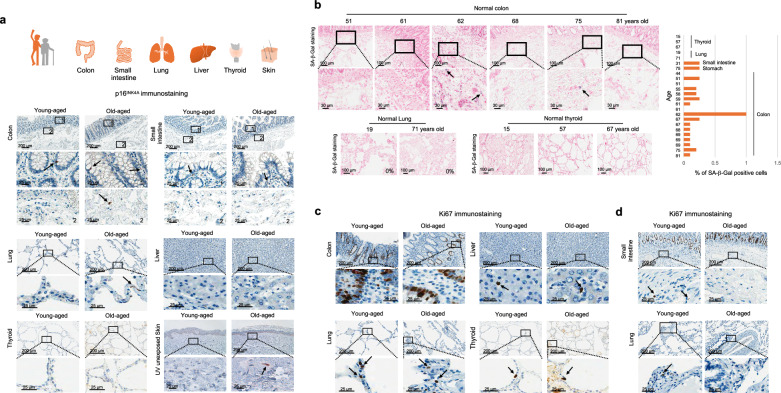
The distribution of p16^INK4A+^ cells in normal tissues from young and aged individuals. **a** IHC analysis for p16^INK4A^ was performed in young and aged human tissues. High-magnification views of the original figure are shown as “1,” “2.” The arrow indicates p16^INK4A+^ cells. **b** SA-β-Gal staining was performed on colon, small intestine, lung, and thyroid tissues from different age groups (left panel). The right panel displays the quantification data of SA-β-Gal^+^ cells. **c** Representative images are Ki67 IHC analysis in tissues from young and elderly subjects. The arrow indicates Ki67-stained cells. **d** Representative images of Ki67-stained stromal regions in human small intestine and lung tissues. The arrow indicates Ki67-stained cells.

**Fig. 2:**
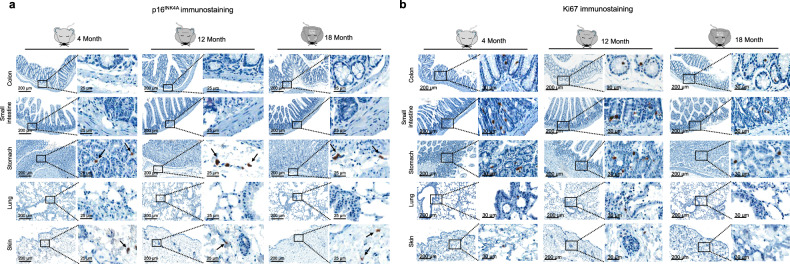
The distribution of p16^INK4A+^ cells in normal tissues from young and aged mice. **a** IHC analysis for p16^INK4A^ in male mouse tissues at 4, 12, and 18 months of age. The arrow indicates p16^INK4A^-stained cells. **b** IHC analysis for Ki67 in male mouse tissues at 4, 12, and 18 months of age.

Although p16^INK4A^ may not be an infallible marker for detecting all senescent cells, p16^INK4A^ is considered the most reliable marker currently available for detecting the later stages of cellular senescence^31^. Our previous study revealed that other senescence markers, such as p21^Waf1^ and p53, encompass a wider range of damaged cells rather than exclusively senescent cells^18^. Therefore, in this study, the quantification of senescent cells in normal tissues primarily relied on the expression of p16^INK4A^ to accurately identify cells in the later stages of the senescence process (p53^+^/ p21^Waf1+^/p16^INK4A+^ cells, hereinafter referred to as fully senescent cells)^18^. We observed that the proportion of p16^INK4A+^ cells was rare (less than 1%) in both the parenchyma and stroma of various tissues from elderly subjects, including the colon, small intestine, lung, unexposed skin, liver, and thyroid (Fig. 1a, Supplementary Fig. 1a, and Supplementary Table 1–7). To confirm the later stage of the senescent phenotype in p16^INK4A+^ cells, we quantified mRNA expression levels of *IFNA1* (IFNα) and *IFNB1* (IFNβ) across three different stages of the senescence process: young (p53^−^/p21^Waf1−^/p16^INK4A−^), intermediate stage of senescence (p53^+^/p21^Waf1+^/p16^INK4A-^), and fully senescent (p53^+^/p21^Waf1+^/p16^INK4A+^)^18^. IFNα and IFNβ are markers indicative of the later stages of cellular senescence^32^. Interestingly, the expression level of *IFNA1* is highly upregulated in the fully senescent cells. However, *IFNB1* continues to be highly expressed throughout the senescence process (Supplementary Fig. 1b–d). To validate the observed scarcity of senescent cells in the elderly’s tissues, other representative senescence markers were assessed. The H3K9me3 IHC analysis showed that there is no significant increase in H3K9me3 in elderly’s tissues (Supplementary Fig. 1e). However, p21^Waf1+^ cells were more frequently observed in the stromal region of elderly tissues (Supplementary Fig. 1f). Notably, not all p21^Waf1+^ cells were positive for p16^INK4A^; p21^Waf1^ can be solely upregulated in the early stage of DNA damage and during the senescence process without p16^INK4A^ expression. Indeed, our previous study demonstrated that p21^Waf1^ is a marker for cells in the intermediate stage of the senescence process, rather than fully senescent cells^18^. Therefore, it is highly suggested that the phenotypic changes in the structural stromal cells are due to the presence of cells in the intermediate stage of the senescence process, rather than the accumulation of fully senescent cells. To further validate the presence of senescent cells in the tissues from the elderly, we conducted senescence-associated β-galactosidase (SA-β-Gal) staining in fresh frozen normal gastrointestinal (GI) tract, lung, and thyroid tissues. The analysis revealed that SA-β-Gal^+^ cells did not exhibit an increasing tendency with aging, both in parenchyma and stroma (Fig. 1b). Ki67 IHC analysis showed parenchymal cells in the elderly’s tissues continued to proliferate despite of the decreased number of Ki67^+^ cells (Fig. 1c, d and Supplementary Fig. 2). Multiplex IHC analysis clearly demonstrated that cells positive for p16^INK4A^ did not exhibit Ki67 positivity (Supplementary Fig. 3). We further confirmed p16^INK4A^ IHC analysis in mice tissues (4, 12, and 18 months). Organs from old-aged mice exhibited similar phenotypes to those of human subjects, as p16^INK4A+^ parenchymal and stromal cells were rarely present (Fig. 2a and Supplementary Fig. 4), and Ki67 staining revealed sustained proliferation of parenchymal cells (Fig. 2b).

### scRNA-seq of normal tissues across different age groups

**Fig. 3:**
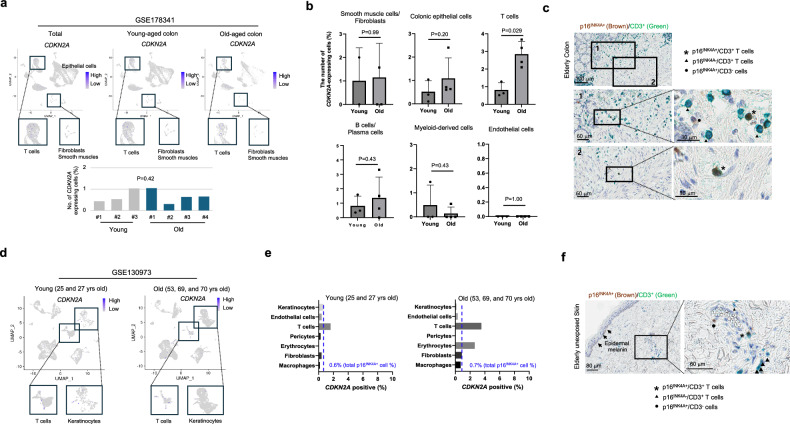
The distribution of *CDKN2A*^+^ cells in normal colon and skin tissues from young and aged individuals in scRNA-seq. **a** Feature plots display the expression of *CDKN2A* in the public dataset of colon, GSE178341 (upper panel). Lower panel shows the proportion of *CDKN2A*^+^ cells in young and elderly individuals, respectively. **b** Bar graphs depict the proportion of *CDKN2A*^+^ cells in each cell type. The graph shows the mean + standard deviation. The *p*-value is obtained using the Mann–Whitney U test. “Young” and “Old” indicate the young and the elderly individuals, respectively. **c** The multiplex IHC analysis shows each subtype of CD3^+^ T cells according to p16^INK4A^ expression. **d** Feature plots display the expression of *CDKN2A* in the public dataset of skin, GSE130973. **e** The proportion of *CDKN2A*^+^ cells in each cell type of skin are shown. **f** The multiplex IHC analysis shows each subtype of CD3^+^ T cells according to the p16^INK4A^ expression. The arrows indicate the epidermal melanin, not immune-positive cells.

**Fig. 4:**
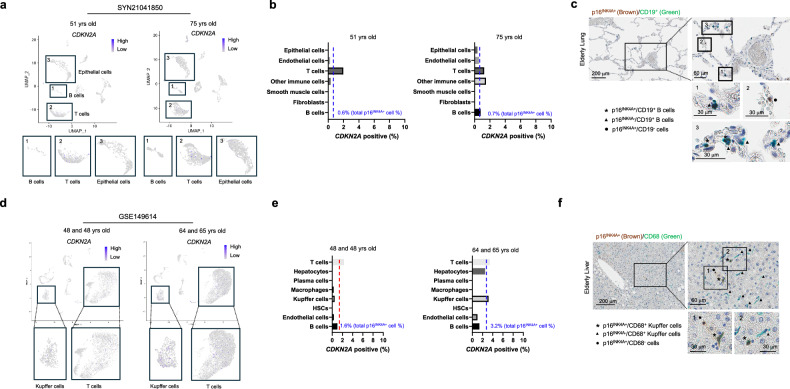
The distribution of *CDKN2A*^+^ cells in normal lung and liver tissues from young and aged individuals in scRNA-seq. **a** Feature plots display the expression of *CDKN2A* in the public dataset of lung, SYN1041850. **b** Bar graphs depict the proportion of *CDKN2A*^+^ cells in each cell type of the lung tissue. **c** The multiplex IHC analysis shows each subtype of CD19^+^ B cells according to the p16^INK4A^ expression. **d** Feature plots display the expression of *CDKN2A* in the public dataset of liver, GSE149614. **e** The quantification data of *CDKN2A*^+^ cells in each cell type of liver are shown. **f** The multiplex IHC analysis shows each subtype of CD68^+^ Kupffer cells according to the p16^INK4A^ expression.

Although there was no statistically significant difference in the number of p16^INK4A+^ cells between tissues from young and elderly individuals, we observed a tendency for an increased proportion of p16^INK4A+^ cells within the stroma compared to the parenchyma (Supplementary Fig. 1). Recognizing that stromal cells consist of both structural stromal cells and immune cells, we hypothesized that the coexistence of structural stromal cells and immune cells in the organic stroma might obscure statistical significance. Therefore, we further analyzed the distribution of p16^INK4A+^ senescent cells using publicly available single-cell RNA-sequencing (scRNA-seq) datasets from various organs. This analysis aimed to detail the distribution of p16^INK4A+^ cells according to cellular subtypes in both humans and mice. When comparing colon tissues between young and elderly individuals (GSE178341)^22^, the expression of the senescence marker p16^INK4A^ was generally around or less than 1% in all individuals across the age (Fig. 3a and Supplementary Fig. 5a). Upon further analysis, no significant differences were observed in colonic epithelial cells, fibroblasts, smooth muscle, or endothelial cells. However, a notable increase in the proportion of p16^INK4A+^ cells was observed in immune cells, especially T cells in elderly tissues (Fig. 3b and Supplementary Fig. 5b). Confirmation through multiplex IHC analysis revealed CD3/p16^INK4A^ double-positive cells were frequently observed in the colon stroma of elderly’s tissues (Fig. 3c). We further analyzed another dataset of skin, lung, and liver in human. The analysis of skin tissues (GSE130973)^23^ revealed that the proportion of p16^INK4A^-expressing cells was 0.6% in young individuals, and the proportion was increased only by 0.1% in old individuals (Fig. 3d, e, Supplementary Fig. 5c, d). When analyzed on a cell type level, similar to the colon, a significant increase in p16^INK4A^ expression was observed in T cells in the skin tissues (Fig. 3f). In normal lung tissues (SYN21041850)^24^, the expression of p16^INK4A^ in individuals aged 75 showed only a marginal increase of approximately 0.1% compared to tissues from 51-year-old individuals (Fig.4a, b, Supplementary Fig. 6a, b). In this case, a higher prevalence of senescent cells was observed in B cells rather than T cells (Fig. 4b, c). Liver (GSE149614)^25^ also showed that a similar trend with a notable increase primarily in immune cells, including T cells and Kupffer cells (Fig. 4d–f and Supplementary Fig. 6c, d). These finding suggested that although the major population of senescent cells, marked by p16^INK4A^, varied depending on the type of the organ, the main increased population of p16^INK4A+^ cells derived from immune system.

**Fig. 5:**
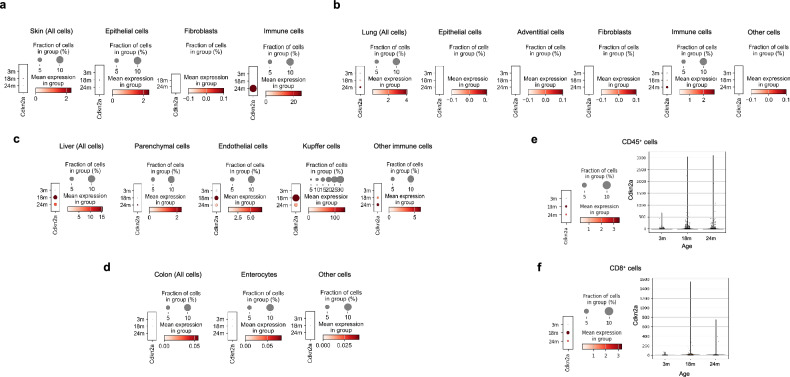
The distribution of *Cdkn2a*^+^ cells in normal tissues from young and aged mice in single-cell RNA-sequencing. **a**–**d** Dot plots display the expression of *Cdkn2a* in cell types on skin, lung, liver, and colon tissues in 3-month (3 m), 18-month (18 m), and 24-month-old (24 m) mice, respectively. **e** A dot plot (left panel) and a violin plot (right panel) shows the expression level and the proportion of *Cdkn2a* expressing cells among CD45^+^ immune cells in 3 m, 18 m, and 24 m mice, respectively. **f** A dot plot (left panel) and a violin plot (right panel) shows the expression level and the proportion of *Cdkn2a* expressing cells among CD8^+^ immune cells in 3 m, 18 m, and 24 m mice, respectively. The size of each dot represents the proportion of cells expressing certain gene in each group (%).

We examined the distribution of p16^Ink4a+^ (*Cdkn2a*^+^) senescent cells in mice (GSE109774)^29^. Analysis revealed elevated levels in the liver and tongue mainly, with some increase in senescent cells observed in the pancreas, skin, heart, and thymus. On the other hand, some organs including bladder and aorta showed decrease in p16^Ink4a+^ cell population (Supplementary Fig. 7a, b). However, when analyzing the entire population of mouse epithelial cells and fibroblasts in major organs including lung, colon, skin and liver, a significant increase was not observed, except liver (Supplementary Fig. 8a–c). These results suggest that the increased p16^INK4+^ cell population identified in mice are mainly neither parenchymal cells nor structural stromal cells. Subsequently, we performed scRNA-seq of p16^Ink4a+^ senescent cells in the lung, liver, colon, and skin. Similar to human tissues, immune cells senescence was observed in the skin, while senescent cells were scarcely observed in fibroblasts and keratinocytes (Fig. 5a and Supplementary Fig. 9a). In the case of lung, an increase in senescent cells was observed in 24-month-old mice, particularly in immune cells rather than fibroblasts or epithelial cells (Fig. 5b, Supplementary Fig. 9b). In the liver, the majority of senescent cells were observed in Kupffer cells, while no significant increase in senescent cells was observed in the colon (Fig. 5c, d and Supplementary Fig. 9c, d). These findings suggest that, similar to elderly humans, fully senescent parenchymal and structural stromal cells scarcely exist in mice. When measuring the expression of p16^Ink4a^ in Cd45^+^ cells of mouse tissues, a significant increase in expression was observed in 18 and 24-month-old mice compared to 3-month-old mice. This increase in p16^Ink4a^ expression in aged mice was also observed when measured in Cd8^+^ cells (Fig. 5e, f). It suggests infiltration of senescent immune cells increases with age in both human and mouse.

### Interaction between senescent T cells and epithelial cells is upregulated in the elderly tissue

**Fig. 6:**
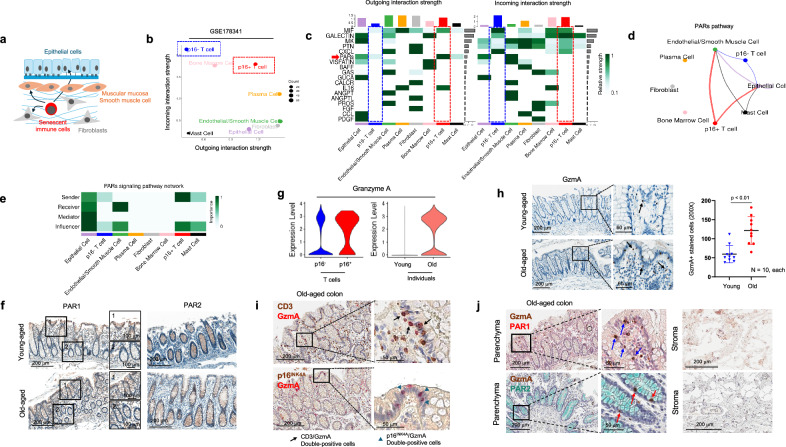
Immune-epithelial interaction in aged colon tissues. **a** The schematic image depicts the effect of senescent immune cells on the surrounding microenvironment of tissues. **b** A dot plot illustrates the incoming and outgoing strength (interaction count) in each cell type in GSE178341. **c** The ingoing and outgoing interaction strength across 18 different signaling pathways in each cell type. A blue box indicates the interaction strength of p16^INK4A-^ T cells, while a red box indicates the interaction strength of p16^INK4A+^ T cells. The y axis of the top bar graph indicates the average number of interactions or connections for each cell type within the signaling network. **d** The interaction strength of PARs signaling network is displayed. **e** The strength of sender, receiver, mediator, and influencer in the PARs signaling pathway network were examined in each cell type. **f** The IHC analysis of PAR1 (left panel) and PAR2 (right panel) in normal colon tissues from young and elderly individuals is shown**. g** The violin plot displays the mRNA expression level of GzmA in p16^INK4A-^ and p16^INK4A+^ T cells from GSE178341 (left panel). The violin plot illustrates the mRNA expression of GzmA in T cells from young and old individuals (right panel). **h** The IHC analysis of GzmA was performed in colon tissues from young and old individuals, respectively (left panel). The right panel shows the quantification data. The data is presented as mean ± standard deviation. “Young” and “Old” indicate the young and the elderly individuals, respectively. The *p*-value is calculated using Mann–Whitney U test. **i** The multiplex IHC analysis shows the expression of CD3 (brown) and GzmA (red) (upper panel) and p16^INK4A^ (brown) and GzmA (red) (lower panel) in old individuals, respectively. **j** The multiplex IHC analysis shows the expression of GzmA (brown) and PAR1 (red) (upper panel) and GzmA (brown) and PAR2 (green) (lower panel) in old individuals, respectively.

Based on the findings above, there was no statistical difference between the fraction of p16^INK4A+^ positivity in parenchymal cells and structural stromal cells in the elderly. However, immune cells demonstrated higher p16^INK4A^ expression in the organs of the elderly, as shown by scRNA-seq and IHC analysis. Given the evident impairment in the functionality of organs and tissues in the elderly, we hypothesized that senescent immune cells accumulating in the organs of the elderly may influence the functionality of epithelial cells, contributing to the observed decline in organ function associated with aging (Fig. 6a). We focused our research on senescent T cells as their p16^INK4A^ expression was significantly and frequently observed in multiple organs, especially in colon. To investigate the cell-cell interactions, we conducted a study on the cell-cell interaction analysis based on mRNA expression using the CellChat package in R^28^. Interestingly, p16^INK4A+^ T cells exhibited distinct characteristics compared to p16^INK4A-^ T cells; p16^INK4A+^ T cells were giving much higher outgoing signals than p16^INK4A-^ T cells (Fig. 6b). It suggests senescent T cells might have a higher capacity to modulate adjacent microenvironment. To unveil the specific ligand-receptor interaction between p16^INK4A+^ T cells and other cell types, the heatmap showing outgoing and incoming signals based on eighteen different signaling pathways (Fig. 6c). The results showed a significant increase in macrophage migration inhibitory factor (MIF), interleukin 16 (IL16), and protease-activated receptor (PAR)-related signaling pathways in p16^INK4A+^ T cells. Among these, MIF and IL16 signaling pathways are mainly upregulated during interactions between immune cells (Supplementary Fig. 10). However, PARs signaling was observed as the main upregulated pathway that senescent T cells affect in parenchymal cells, especially colonic epithelial cells (Fig. 6d). Specifically, p16^INK4A+^ T cells were the strongest sender, and epithelial cells were the main receiver of the PAR-related signaling (Fig. 6e). The PAR family consists of four receptors: from PAR1 to PAR4. Previous studies have indicated that colonic epithelial cells mainly express PAR1 and PAR2 under normal physiological conditions^33^. IHC analysis showed marked expression of PAR1 and PAR2 in normal epithelial cells regardless of the age (Fig. 6f). Several secretory factors identified to activate the PARs signal have been found to be predominantly proteases, such as granzyme A (GzmA) and serine protease 3^34,35^. Among these, GzmA was identified as a substance highly secreted by senescent T cells, which can activate PARs (Fig. 6g). The mRNA expression of GzmA was increased in p16^INK4A+^ T cells, as well as in the T cells of aged tissues (Fig. 6h), however, the expression of serine protease 3 and cathepsin G did not increase significantly or were not expressed in senescent T cells (Supplementary Fig. 11). IHC analysis showed a marked increase in the number of GzmA^+^ T cells in elderly tissues (Fig. 6i). The multiplex IHC analysis revealed that the major population of GzmA is CD3^+^ T cells, and p16^INK4A+^ round-shaped cells mainly express GzmA (Fig. 6i). Moreover, the multiplex IHC analysis suggested that GzmA^+^ T cells were located near PAR1 and PAR2-high colonic epithelial cells, indicating potential interaction between the two cell types, and the interaction between the cell types was rarely observed in the stroma (Fig. 6j).

### GzmA from senescent T cells can lead to epithelial dysfunction in the colons of aged individuals

**Fig. 7:**
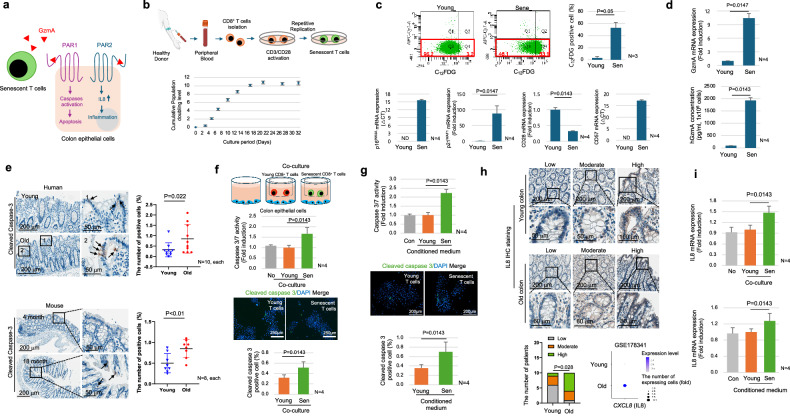
The interaction between senescent T cells and epithelial cells in vitro. **a** A schematic image illustrating how GzmA affects colonic epithelial cells through PARs signaling. **b** Overall scheme of the T cell isolation and senescence process (upper panel). Isolated pan-T cells after stimulation with anti-CD3/28 cocktail and rhIL-2 were assessed for cumulative population doubling level (PDL) (lower panel). Young cells were defined as 2 < cPDL < 5 and senescent cells as cPDL > 9. **c** The C_12_FDG staining analysis using flow cytometry (upper panels). The mRNA expression level of representative T cell senescence markers (p21^Waf1^, p16^INK4A^, CD28, and CD57) are shown (lower panel). ND not detected. **d** The mRNA (upper panel) and protein (lower panel) levels of GzmA in young and senescent T cells are performed by real-time PCR and ELISA, respectively. **e** The IHC analysis of cleaved caspase-3 in normal colon tissues from young and elderly individuals (upper panel) and 4-month and 18-month-old mouse colon tissues (lower panel). The right panels display the quantification data, respectively. **f**,**g** Human primary colon epithelial cells were co-cultured with young or senescence T cells (**f**) or treated with CM of young or senescence T cells (**g**) for 3 days. The luminescence-based assay and ICC to determine cleaved caspase-3/7 activity and cleaved caspase-3 expression level were performed, respectively. **h** IHC analysis for IL8 was performed in normal colon from young and elderly tissues (upper panel). The IHC analysis results are categorized into “Low,” “Moderate,” and “High” based on the intensity of the immunostaining, with representative images provided for each category. The left lower panel shows the quantification data for IHC analysis. The right lower panel shows a dot plot illustrating the expression of *CXCL8* (IL8) in colonic epithelial cells from young and elderly individuals in the GSE178341 data set. **i** The *CXCL8* (IL8) mRNA expression was analyzed by real-time PCR in human colon epithelial cells, which were co-cultured with young or senescent T cells (upper panel) and treated with CM of young or senescent T cells (lower panel) for 3 days. A *p*-value in (**h**) is calculated using the Chi-square test. The rest of the *p*-values are calculated using the Mann–Whitney U test. The graph in (**b**) is shown as mean ± standard deviation, while the rest of the bar graphs are shown as mean + standard deviation. “Young” and “Sen” in (**c**,**d**,**f**,**g**,**i**) indicate the young and senescent T cells, respectively. “Young” and “Old” in (**e**,**h**) indicate the young and the elderly individuals, respectively.

PARs have been reported to undergo N-terminal cleavage by GzmA, leading to their activation and involvement in various cellular processes, notably inflammation and apoptosis^36,37^. Specifically, PAR1 and PAR2 are expressed in gut epithelial cells among a PARs family. PAR1 is associated with the apoptotic signaling pathway, and PAR2 is linked to inflammation, with PAR2 expression well-established in inflammatory bowel diseases^37,38^ (Fig. 7a). Considering that GzmA expression is highly increased in senescent T cells based on scRNA-seq data, we assessed apoptosis and inflammation in the colons of elderly individuals in relation to the PARs signaling pathway, and we established an in vitro senescent T cell model to elucidate the role of senescent T cells on colonic epithelial cells.

Peripheral blood T cells were isolated and stimulated with CD3/CD28 beads, and finally underwent serial passaging resulting in the generation of senescent T cells (Fig. 7b). Their senescent phenotype was confirmed through the expression of markers such as p16^INK4A^, p21^Waf1^, C_12_FDG, CD28, and CD57 (Fig. 7c). Notably, in vitro senescent T cells exhibited elevated expression of GzmA both in RNA and protein levels, consistent with observations in vivo (Fig. 7d). To observe the activation of PARs signaling, we examined the downstream phenotypes of PAR1 and PAR2, specifically apoptosis and inflammation, respectively in vivo. Our results showed a significant increase in cleaved caspase-3^+^ epithelial cells in the colon tissues of elderly individuals and aged mice (Fig. 7e). To validate this phenotype in vitro, we co-cultured senescent T cells with colon epithelial cells and observed an increase in caspase 3/7 activity and cleaved caspase-3 protein levels (Fig. 7f). Furthermore, CM from senescent CD8^+^ T cells also increased caspase 3/7 activity and cleaved caspase-3 protein levels in primary colon epithelial cells (Fig. 7g). We further validated the effect of GzmA on the apoptotic cell death of colonic epithelial cells, recombinant human GzmA (rhGzmA) was applied, which resulted in increased caspase 3/7 activity. Subsequently, treating with PAR1 antagonist reduced the caspase 3/7 activity (Supplementary Fig. 12). GzmA inhibition in conditioned media from senescent T cells, using nafamostat mesylate (NM), a known GzmA inhibitor^39^, resulted in decreased caspase-3/7 activity in colonic epithelial cells (Supplementary Fig. 13).

**Fig. 8:**
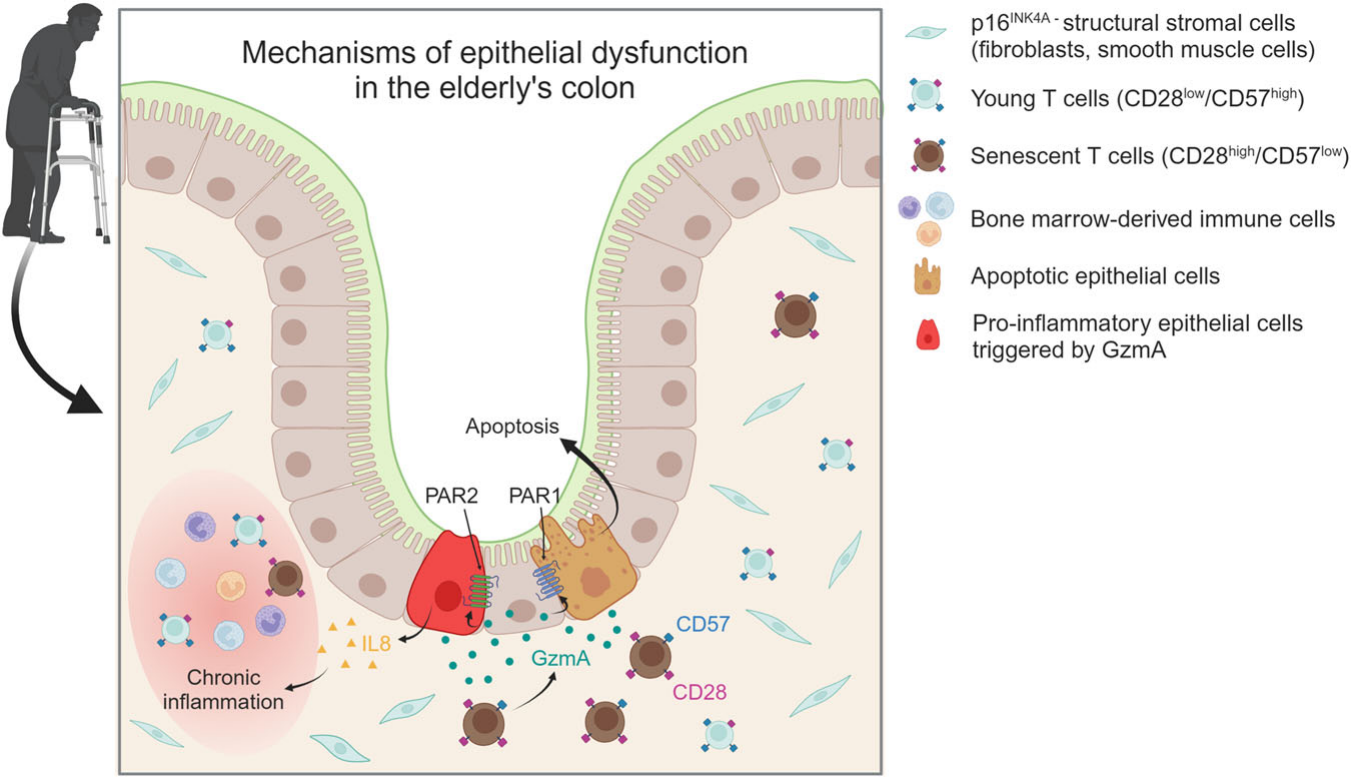
Mechanisms of epithelial dysfunction in the elderly’s colon. A schematic image illustrating how senescent T cells can affect colonic epithelial function *via* the PARs signaling pathway.

The following study investigated GzmA-PAR2 signaling. PAR2 signaling is primarily known to be involved in inflammatory processes^40,41^. A well-known indicator of PAR2-activated inflammation downstream in inflammatory bowel diseases is IL8 (*CXCL8*)^42,43^. Therefore, to assess the PAR2 activation in vivo, IL8 IHC analysis was performed in young and old individuals, and IHC revealed that IL8 expression is significantly increased in the elderly’s tissue (Fig. 7h). In vitro co-culture and CM treatment validated that senescent T cells increase IL8 expression in colonic epithelial cells (Fig. 7i). Specifically, treatment with rhGzmA also increased IL8 expression, indicating that PAR2 signaling is activated. Furthermore, GzmA inhibition in conditioned media from senescent T cells using GzmA inhibitor, resulted in decreased IL8 mRNA expression in colonic epithelial cells (Supplementary Fig. 13 and 14). This suggests that the PAR1 and PAR2 signaling pathways are downregulated by GzmA inhibition. Additionally, other representative inflammatory cytokines were not increased in epithelial cells co-cultured with senescent T cells, suggesting that T cell senescence is not associated with the general inflammation in the elderly colon (Supplementary Fig. 15). These findings suggest a synergistic effect between the apoptotic signaling pathway *via* PAR1 and the chronic inflammatory microenvironment *via* PAR2 (Fig. 8). They also highlight the potential impact of senescent immune cells on parenchymal cell dysfunction, offering insights into possible therapeutic interventions targeting these interactions.

## Discussion

In this study, we analyzed the distribution of fully senescent cells in the organs of elderly individuals by examining p16^INK4A^ expression and SA-β-Gal staining. Despite ongoing controversy regarding p16^INK4A^ expression as a surrogate marker for in vivo cellular senescence, INK-ATTAC and p16-3MR transgenic mouse models clearly showed declines in aging-associated p16^INK4A+^ cells following pharmacological treatment^7,44^. Notably, Omori and colleagues generated a p16^INK4A^-tdTomato reporter mouse and analyzed various organs of 2- and 7-month-old mice^3^. They observed an overall increase in tdTomato^+^ cells in tissues from 7-month-old mice across various organs in both immunofluorescence (IF) staining and scRNA-seq; however, exact cell type annotation of tdTomato^+^ cells were performed only in scRNA-seq, and evidence from IF or IHC is lacking. Additionally, Grosse and colleagues performed a study using a p16-CRE/R26-mTmG mouse to reveal that CD31^+^ endothelial cells and F4/80^+^ macrophages comprise the major population of p16^INK4A+^ cells in the liver^13^. However, information about the remaining population of p16^INK4A+^ cells and other organs is also lacking. Despite the differences, one common finding in the above studies is that the major population of p16^INK4A+^ cells were stromal cells rather than parenchymal cells, which aligns with our current study. Moreover, in our previous study, we found that fibroblasts and smooth muscle cells, which are neither young nor fully senescent but instead are in an intermediate state of senescence, may contribute to another aspect of the aging process^18^. These cells can accumulate and have detrimental effects on the functional integrity of parenchymal cells in the tissues of the elderly, which partly explains the functional decline in aged organs. Therefore, it is strongly suggested that the senescence of stromal cells, rather than parenchymal cells, might be a driver of organic senescence in the natural aging process.

Another major finding of this study is the subtle increase in p16^INK4A+^ cells in the organs of elderly individuals compared to those of young individuals. In our study, we did not find statistical differences when we counted p16^INK4A+^ cells in IHC analysis of parenchymal and stromal compartments, respectively. However, detailed analysis dividing the stroma into structural stromal cells and immune cells revealed a subtle increase in immune cells in the tissues of elderly individuals in certain organs. Although there was a numerical increase, it was around 2% or less in most organs. Omori and colleagues, using p16^Ink4a^-tdTomato reporter mice at 2 months and 7 months of age, also showed an increase in tdTomato^+^ cells. The increase was less than 2% in the kidney (0.5% to 1.5%), lung (0.4% to 1.3%), liver (0.5% to 2.4%), and skin (0.2% to 2%) in IF staining of young and aged mice, respectively^3^. Similarly, Grosse and colleagues compared 2-month-old and 1-year-old p16-Cre/R26-mTmG mice and revealed a subtle increase in p16^INK4A+^ endothelial cells in the liver (1.7% to 3.0%)^13^. These results indicate that the increase in the absolute number of fully senescent cells in the elderly is not substantial. Senescent cells can be eliminated by immune cells in normal condition^45^; thus, the presence of senescent cells at a particular time point is determined by the balance between development and immune clearance. This balance may partly explain the scarcity of fully senescent cells. Although our scRNA-seq analysis also indicates the scarcity of fully senescent cells, it is important to note that sample preparation is challenging, and the cell dissociation yield for stromal cells is significantly lower than for parenchymal/epithelial cells. Therefore, the proportion of *CDKN2A*^+^ cells in scRNA-seq data may be easily influenced by the quality of sample preparation^46^. Nevertheless, since the scRNA-seq and IHC data showed similar results, it was considered that p16^INK4A^-positive cells are rare in elderly tissue.

If the parenchymal cells in organs are still functioning and the number of fully senescent cells is minimally increased in the elderly, why is the aging phenotype and functional decline so pronounced in aged individuals? The exact mechanism is still unknown, but there are several potential explanations. [1] Even a small increase in fully senescent cells can significantly affect organ function. Senescent cells are active senders of signals, rather than receivers, and they actively express secreting molecules including SASPs^5^. This means they can easily affect adjacent cells and ECM components even in small numbers. [2] Another point is that we previously reported that the accumulation of cells in the intermediate stage of the senescence process in the elderly’s tissues also can contribute to decrease of parenchymal cell function^18^. [3] Lastly, the decreased function of epithelial or mesenchymal stem cells may affect the overall composition of functioning cells in the organ. Although we could not specify p16^INK4A+^ epithelial and mesenchymal stem cells in our tissue samples, it is possible that the stem cells in elderly tissues are in an intermediate stage of overall stem cell fate. Investigating this possibility could be the focus of a future study. Therefore, we currently recognize that the aged phenotype of elderly individuals might be the synergistic outcome of these factors.

Immunosenescence is the term associated with immune system dysfunction caused by aging^47–49^. Multiple previous studies have supported the notion that mild and chronic inflammation is present in the tissues of elderly individuals. In line with this concept, Franceschi first proposed the term “inflammaging,” referring to highly regulated pro-inflammatory markers in tissues from the elderly^11^. As the senescence of immune cells shares some characteristics with the well-established senescence model of fibroblasts, researchers have begun to establish the hallmarks of immunosenescence^50^. Interestingly, these hallmarks include the accumulation of CD8^+^ memory T cells and a deficit of B cells, which was confirmed in about 15% of 85-year-old individuals in the OCTO/NONA studies^51^. Therefore, immunosenescence primarily focuses on the senescence of T cells. Mittelbrunn and Kroemer established the “Hallmarks of T Cell Aging” in 2021^52^. They suggested ten hallmarks of T cell aging, including thymic involution, naïve-memory imbalance, T cell senescence (halted proliferation of T cells), and inflammaging. Interestingly, our current study’s senescence model of isolated T cells also showed hallmarks of T cell aging, including an increase in CD28, a decrease in CD57 expression, halted proliferation, and increased expression of cytokines, suggesting that our T cell senescence model worked appropriately. Notably, in our model, the expression of representative granzymes, granzyme A and granzyme B, was highly upregulated compared to other proteases that can cleave PARs for signaling activation (Supplementary Fig. 16). This suggests that PAR signaling of epithelial cells in the elderly is specifically activated by granzyme-releasing cells, with senescent T cells potentially being a major driver. These results suggest that senescent T cells can be activated independent of antigen, so called “bystander activation^53^” and weaken the function of surrounding normal cells, which may be closely related to the aging process.

Another interesting question that can be raised is: where is the source of immunosenescence? It is highly suggested that resident and infiltrating immune cells possess different senescence processes, but information on both cell types is still lacking. Moreover, although thymic involution is one of the hallmarks of T cell aging, the specific organ associated with the T cell aging process among T cell-dwelling organs, such as the target organ, spleen, bone marrow, and thymus, is still unknown. However, our IHC analysis of spleen from young and aged individuals showed that there is increase in the number of p16^INK4A+^ T cells in the spleen from aged individuals, and it suggests that T cells were already being senescent before infiltration to target organ (Supplementary Fig. 17).

Conclusively, our results demonstrated that epithelial cells, stromal fibroblasts, and smooth muscle cells in the organs of elderly individuals are not undergoing complete senescence; however, immune cells were observed to undergo senescence. Senescent immune cells were found to induce apoptosis of epithelial cells or trigger inflammation, thereby inhibiting the function of epithelial cells. Collectively, this study proposes a new anti-aging strategy aimed at improving the quality of life in elderly individuals by regulating senescent immune cells to restore organ function.

## Supplementary information

Supplementary Information

Supplementary Data

## Data Availability

The data presented in this study are publicly available in NCBI’s Gene Expression Omnibus (GEO) under the accession codes GSE109774, GSE178341, GSE130973, and GSE149614, and in the UCSC Cell Browser under the accession code SYN21041850.

## Code availability

The R and Python code used in this study is available under a reasonable request to a corresponding author, T.J.P. (park64@ajou.ac.kr).
