# Supplementary material for: Distribution and impact of p16INK4A+ senescent cells in elderly tissues: a focus on senescent immune cell and epithelial dysfunction
Source: Exp Mol Med. 2024 Dec 2;56(12):2631–41. doi: 10.1038/s12276-024-01354-4 (PMC11671602; doi:10.1038/s12276-024-01354-4)
Supplement: Supplementary file 1 — Supplementary Information [file 12276_2024_1354_MOESM1_ESM.pdf]

Supplementary Fig. 1

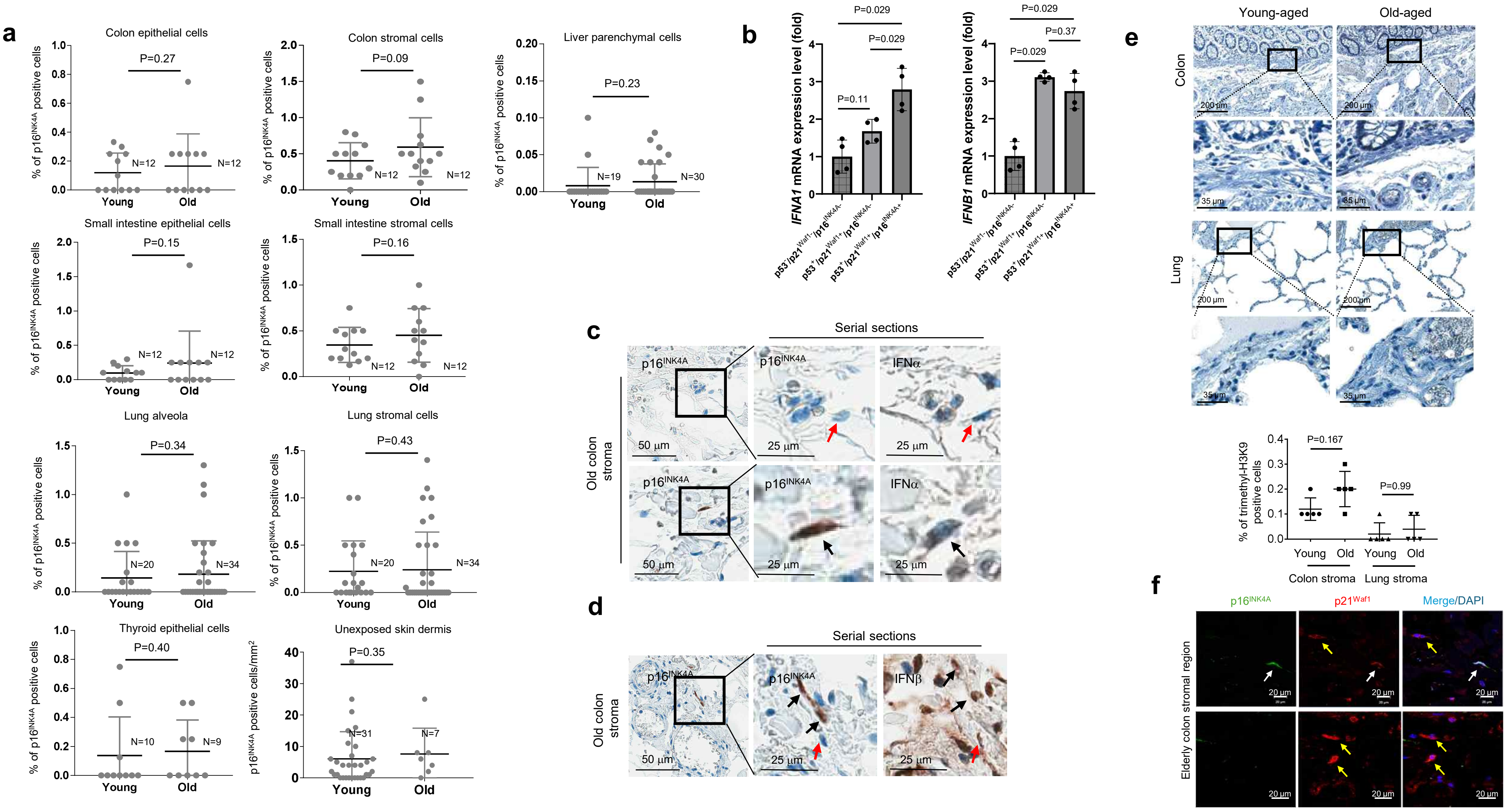

Supplementary Fig. 2

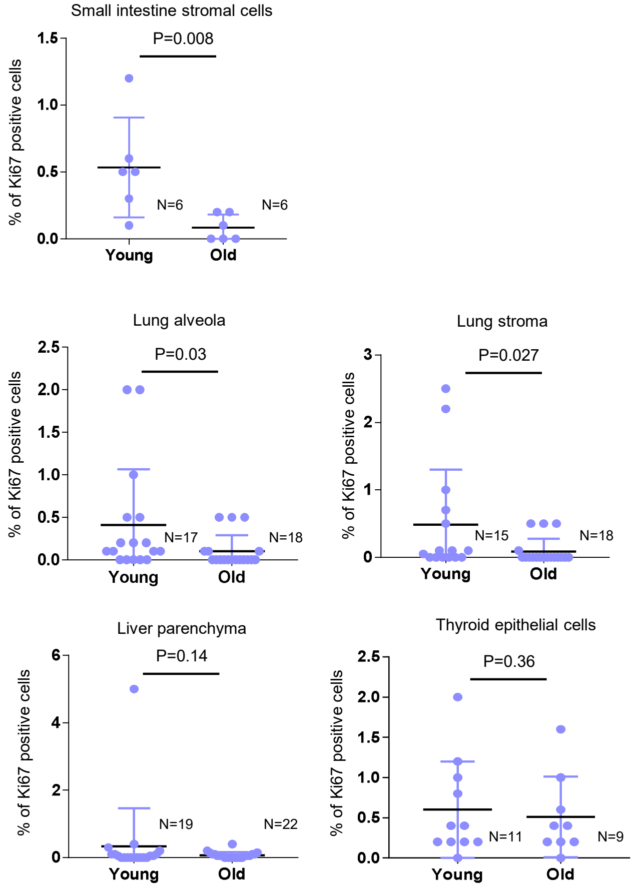

Supplementary Fig. 3

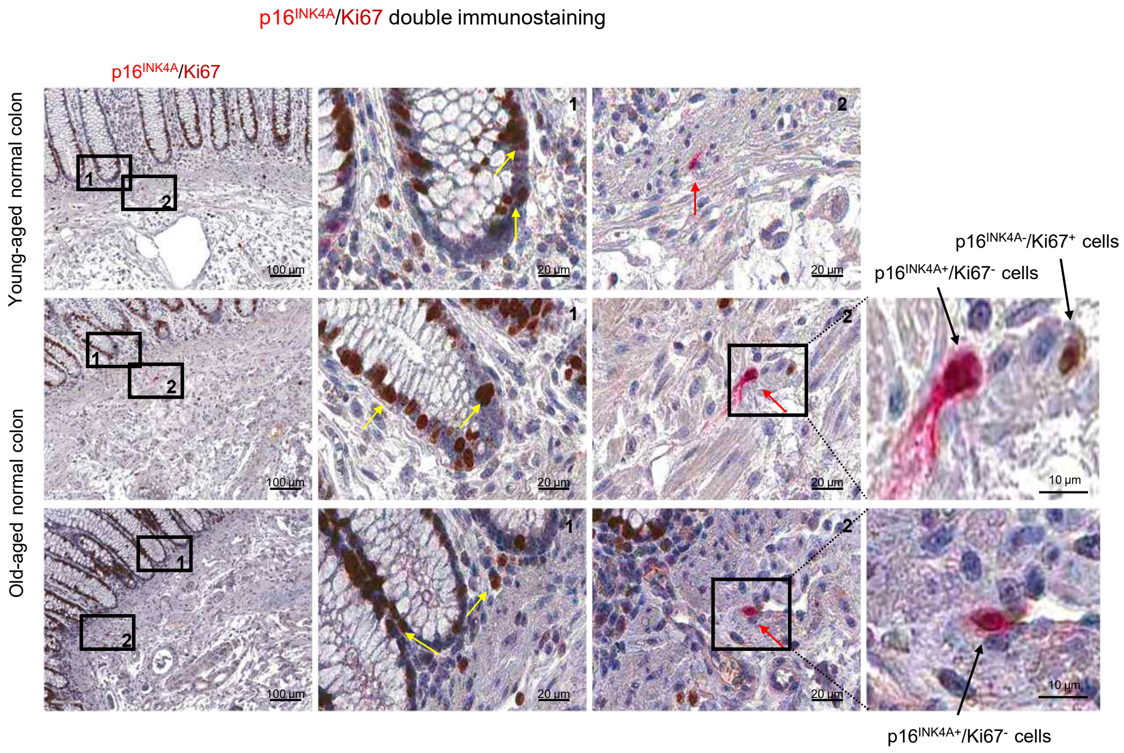

Supplementary Fig. 4

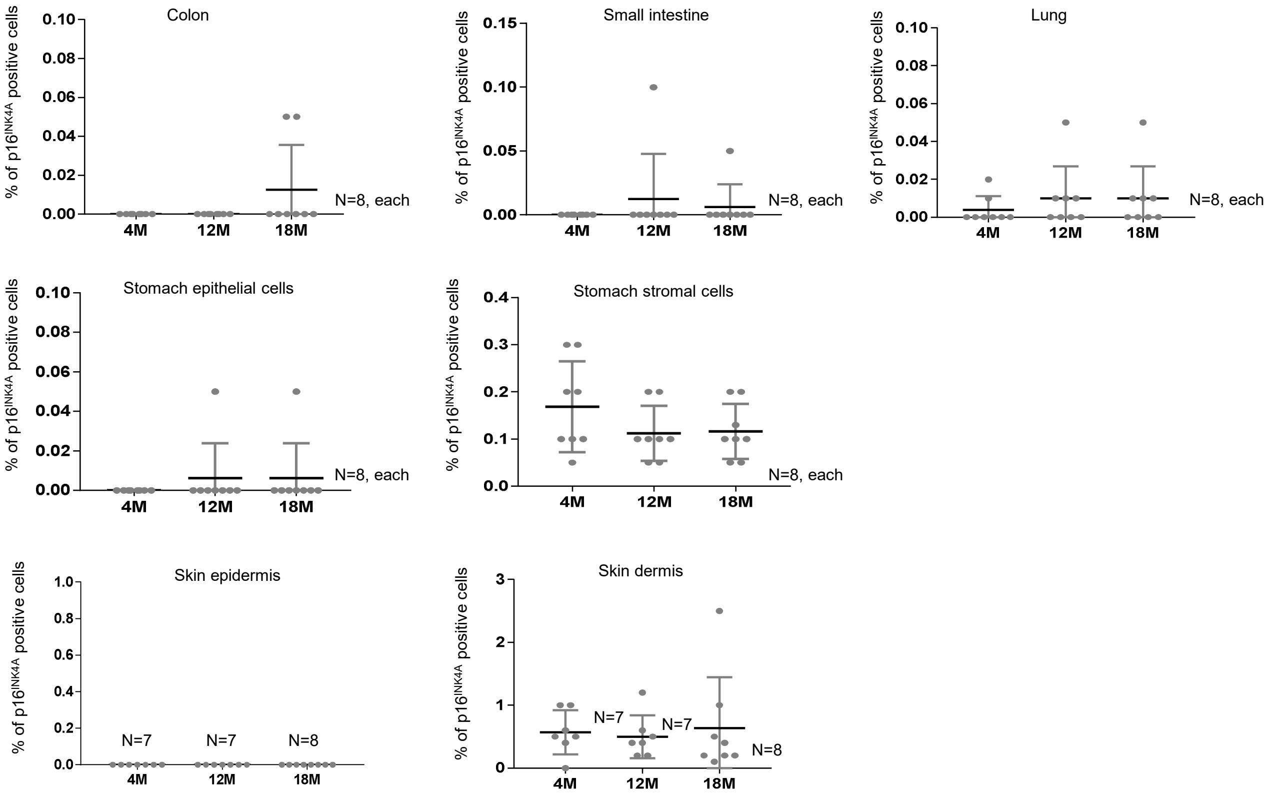

Supplementary Fig. 5

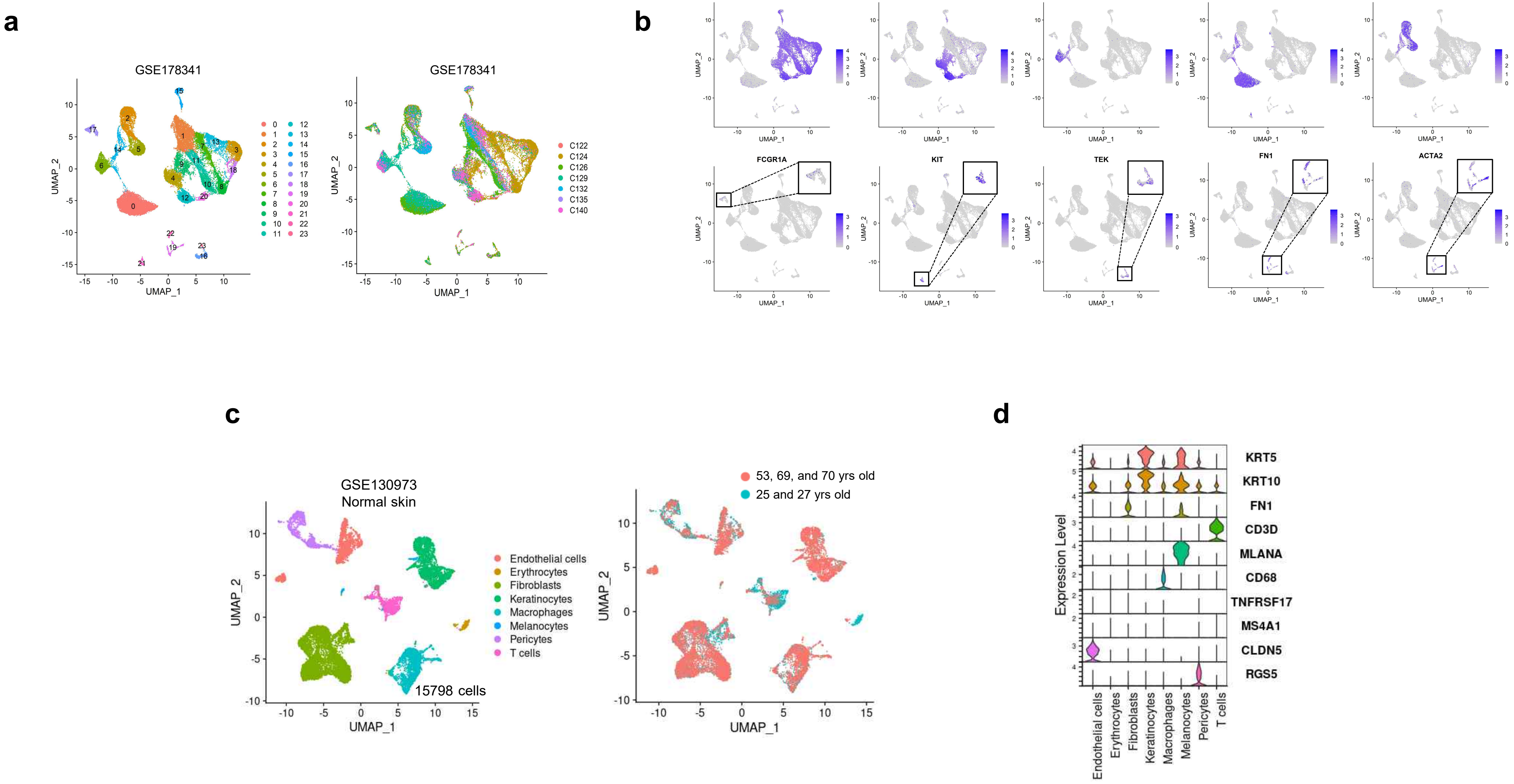

Supplementary Fig. 6

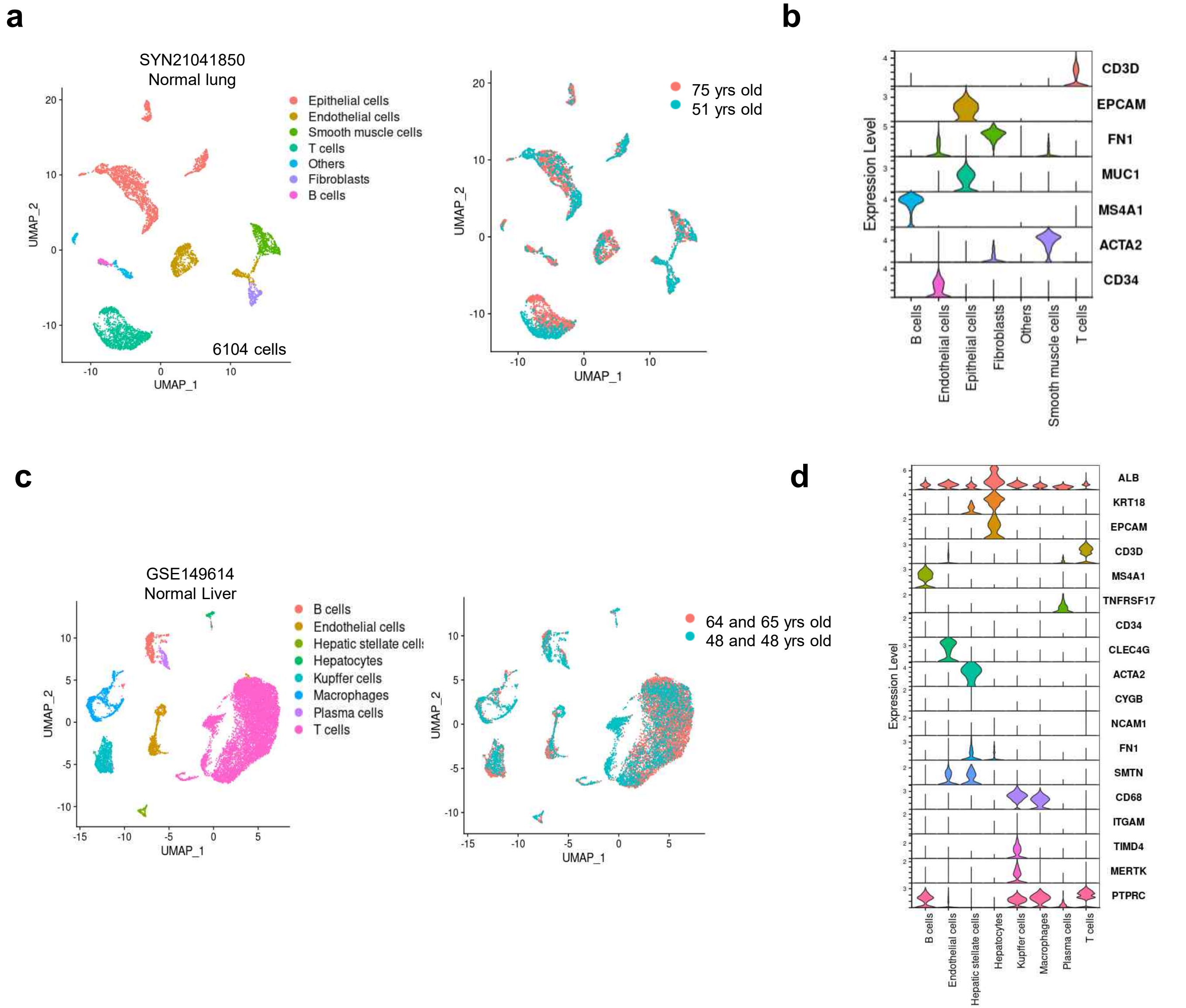

Supplementary Fig. 7

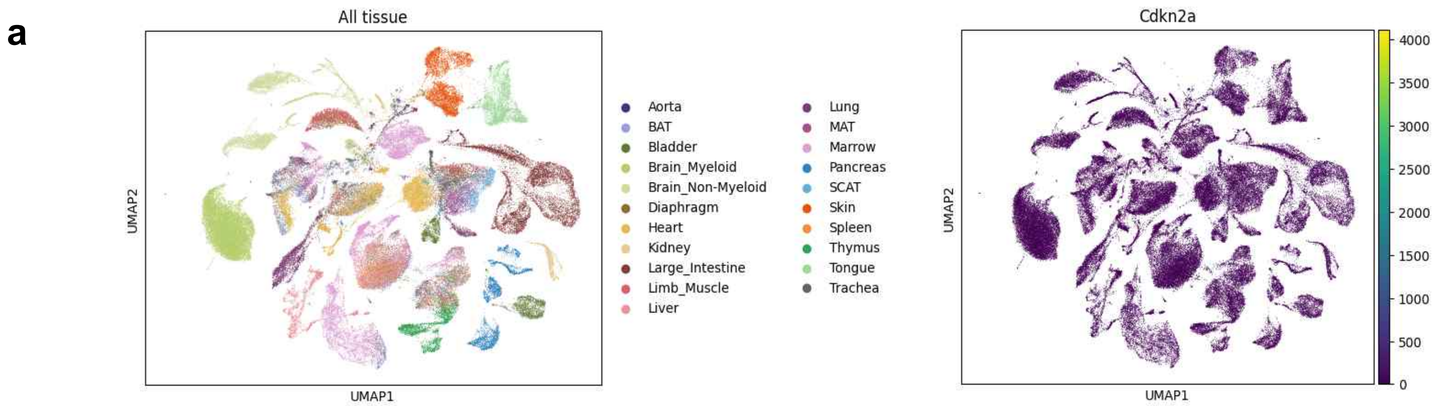

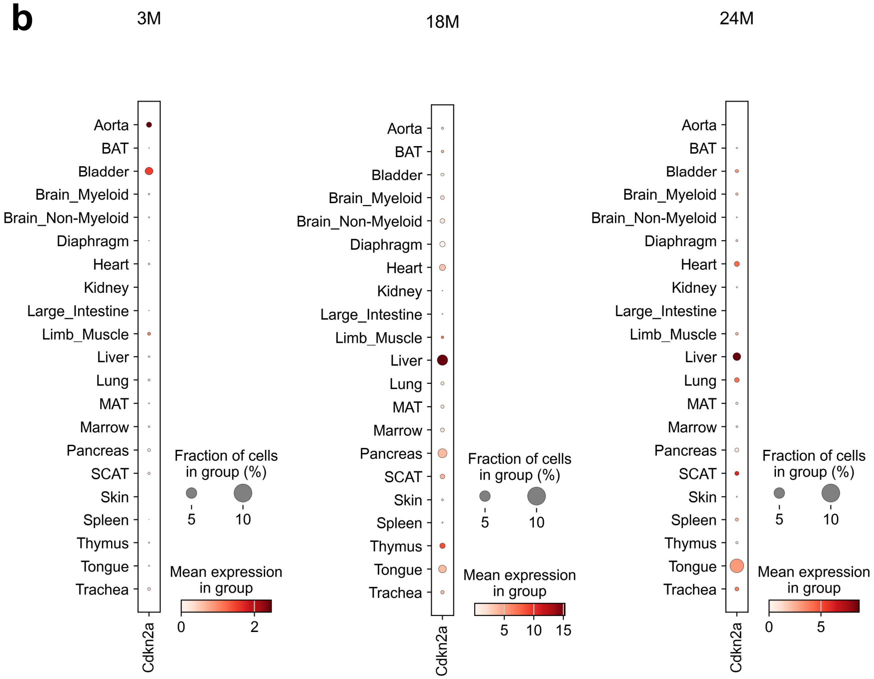

Supplementary Fig. 8

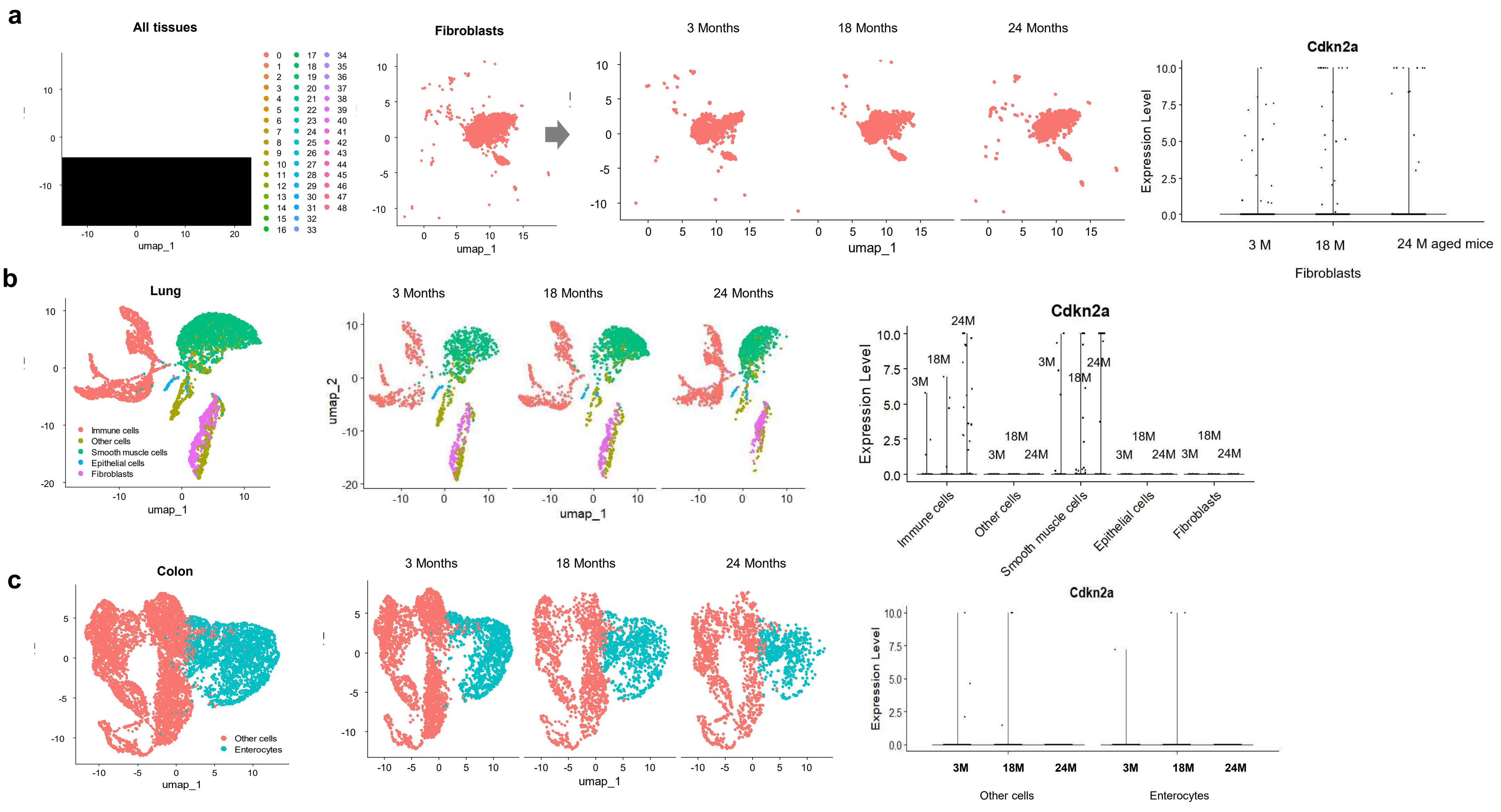

Supplementary Fig. 9

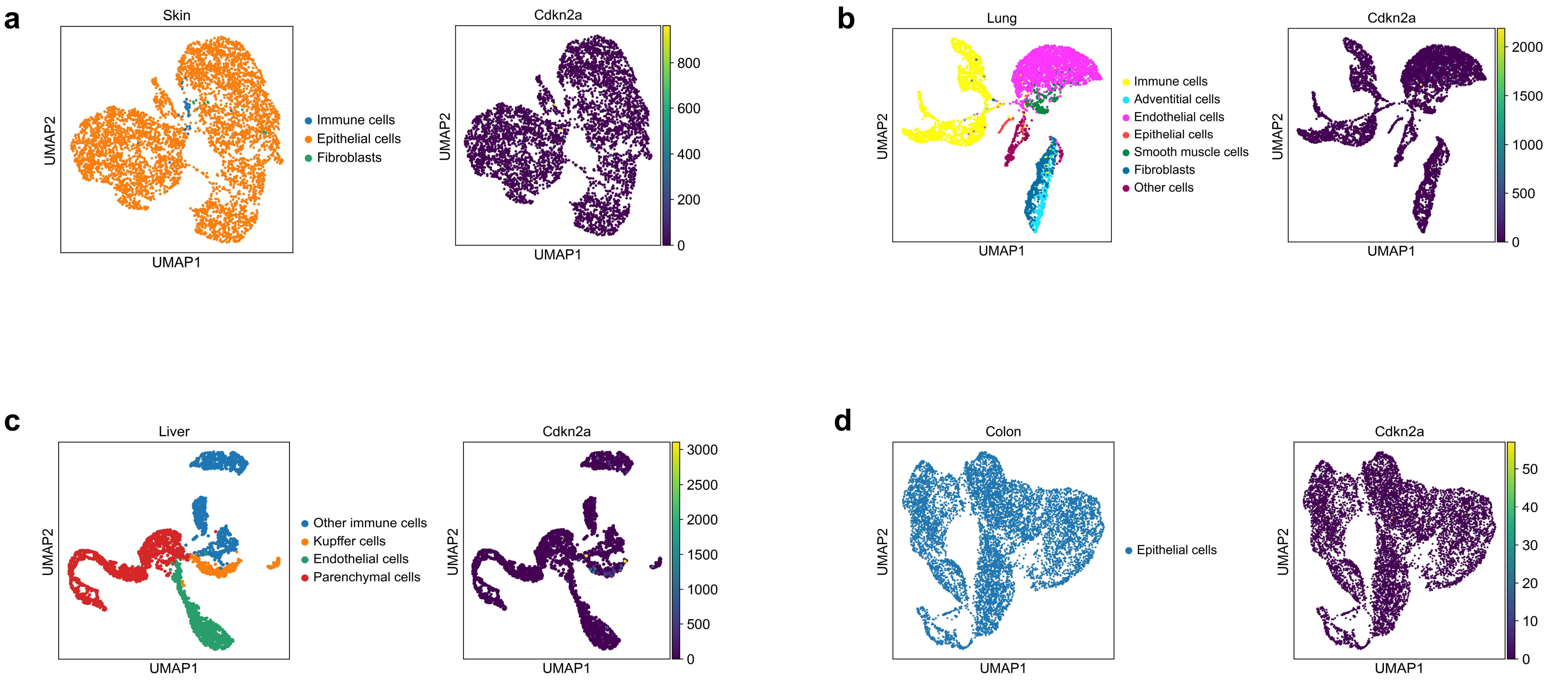

Supplementary Fig. 10

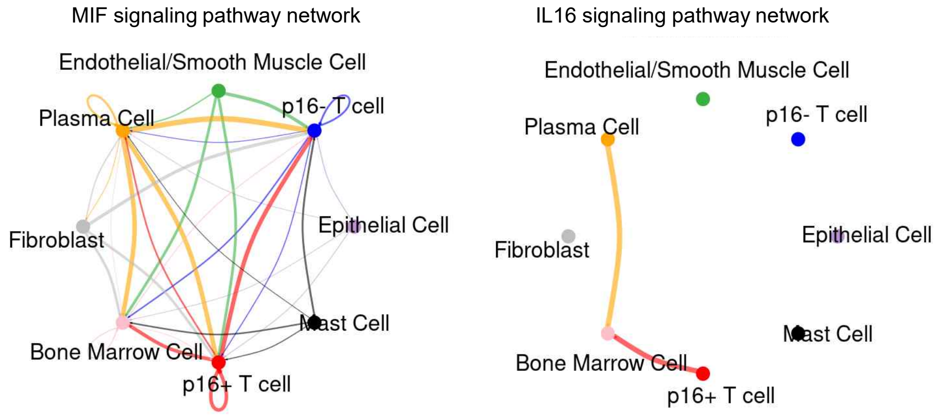

Supplementary Fig. 11

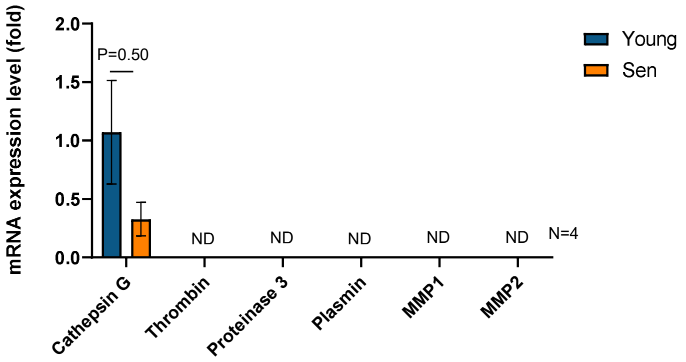

Supplementary Fig. 12

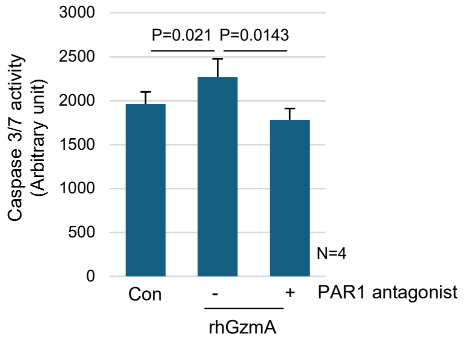

Supplementary Fig. 13

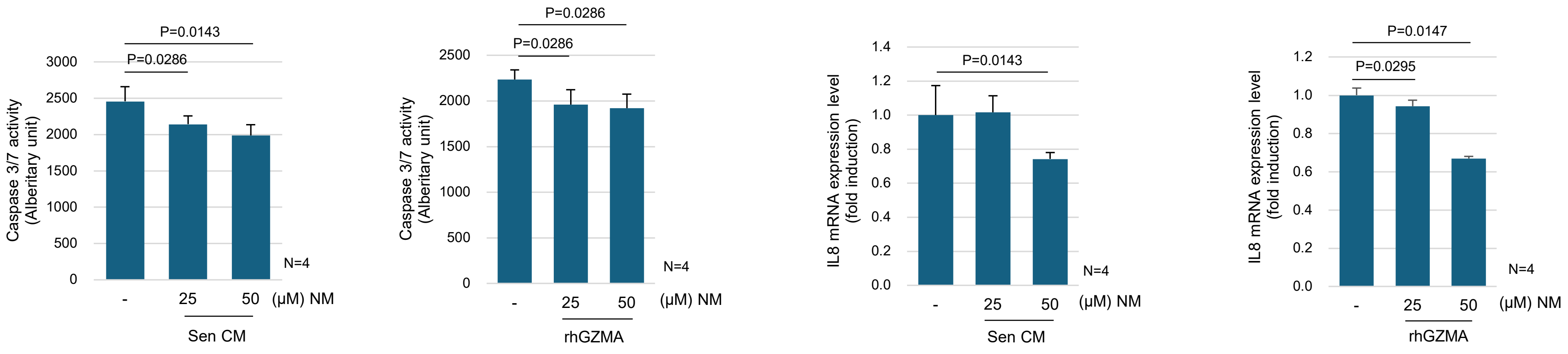

Supplementary Fig. 14

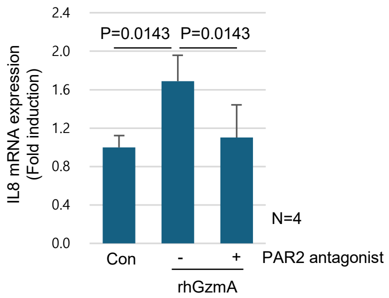

Supplementary Fig. 15

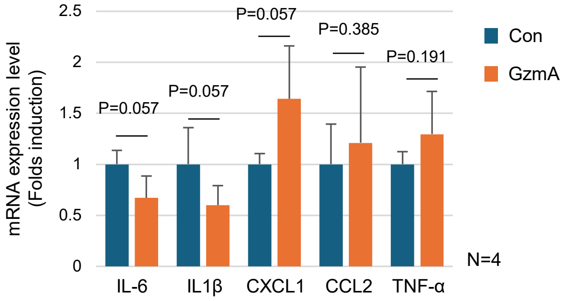

Supplementary Fig. 16

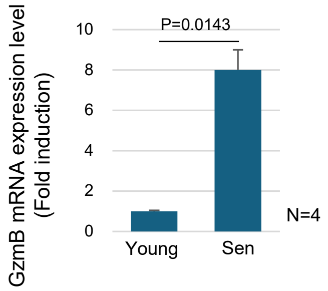

Supplementary Fig. 17

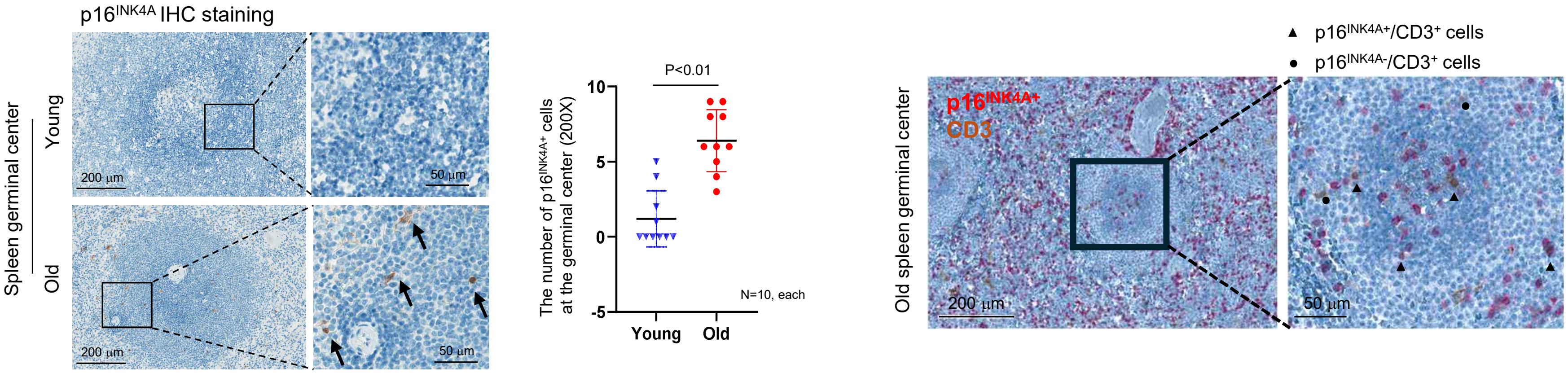

Supplementary Table 1. Patient information of colon donors used in this study.

	Young (n=18)	Old (n=20)	p value
**Sex: Male (%)**	9 (50.0)	12 (60.0)	0.38[a]
**Age: Median [range]**	33.5 [20-40]	82.0 [80-90]	<0.0001[b]
**Diagnosis (%)**			0.0003[a]
**Diverticulum-related diseases**	9 (50.0)	0 (0.0)	
**Tumor**	9 (50.0)	20 (100.0)	

[a]*p* values are obtained using Chi-square test. [b]*p* value is obtained using two-tailed Student's *t* test.

Supplementary Table 2. Patient information of lung donors used in this study.

	Young (n=20)	Old (n=34)	p value
**Sex: Male (%)**	14 (70.0)	19 (55.9)	0.30[a]
**Age: Median [range]**	27 [19-38]	77 [75-83]	<0.0001[b]
**Diagnosis (%)**			<0.0001[a]
**Sequestration**	1 (5.0)	0 (0.0)	
**Bulla**	1 (5.0)	0 (0.0)	
**Pneumothorax**	13 (65.0)	0 (0.0)	
**Tumor**	5 (25.0)	34 (100.0)	

[a]*p* values are obtained using Chi-square test. [b]*p* value is obtained using two-tailed Student's *t* test.

Supplementary Table 3. Patient information of liver donors used in this study.

	Young (n=19)	Old (n=30)	p value
**Sex: Male (%)**	12 (63.2)	14 (46.7)	0.26[a]
**Age: Median [range]**	29 [20-42]	83 [80-87]	<0.0001[b]
**Diagnosis (%)**			<0.0001[a]
**Liver transplant donor**	19 (100.0)	0 (0.0)	
**Tumor**	0 (0.0)	28 (93.4)	
**Bile duct stone**	0 (0.0)	1 (3.3)	
**Fatty change**	0 (0.0)	1 (3.3)	

[a]*p* values are obtained using Chi-square test. [b]*p* value is obtained using two-tailed Student's *t* test.

Supplementary Table 4. Patient information of skin donors used in this study.

	Young (n=31)	Old (n=7)	p value
**Sex: Male (%)**	14 (45.2)	3 (42.9)	0.91[a]
**Age: Median [range]**	19 [3-39]	72 [71-81]	<0.0001[b]
**Diagnosis (%)**			0.29[a]
**Morphea**	3 (9.7)	1 (14.3)	
**Idiopathic Guttate Hypomelanosis**	12 (38.7)	4 (57.1)	
**Linchen striatus**	1 (3.2)	0 (0.0)	
**Nevus**	4 (12.9)	0 (0.0)	
**Bilateral telangiectatic macule**	1 (3.2)	0 (0.0)	
**Atrophoderma**	1 (3.2)	0 (0.0)	
**Lentiginosis**	1 (3.2)	0 (0.0)	
**Confluent and reticulated Papillomatosis**	6 (19.4)	0 (0.0)	
**Acanthosis nigricans**	1 (3.2)	0 (0.0)	
**Café au lait macule**	1 (3.2)	0 (0.0)	
**Livedoid vasculitis**	0 (0.0)	1 (14.3)	
**Steroid-induced atrophy**	0 (0.0)	1 (14.3)	

[a]*p* values are obtained using Chi-square test. [b]*p* value is obtained using two-tailed Student's *t* test.

Supplementary Table 5. Patient information of small intestine donors used in this study.

	Young (n=12)	Old (n=12)	p value
**Sex: Male (%)**	7 (58.3)	7 (58.3)	1.00[a]
**Age: Median [range]**	32.5 [22-39]	83 [80-87]	<0.0001[b]
**Diagnosis (%)**			<0.0001[a]
**Diverticulitis**	6 (50.0)	0 (0.0)	
**Tumor**	6 (50.0)	12 (100.0)	

[a]*p* values are obtained using Chi-square test. [b]*p* value is obtained using two-tailed Student's *t* test.

Supplementary Table 6. Patient information of thyroid donors used in this study.

	Young (n=11)	Old (n=9)	p value
**Sex: Male (%)**	2 (18.2)	1 (11.1)	0.66[a]
**Age: Median [range]**	38 [29-40]	71 [70-87]	<0.0001[b]
**Diagnosis (%)**			1.00[a]
**Tumor**	11 (100.0)	9 (100.0)	

[a]*p* values are obtained using Chi-square test. [b]*p* value is obtained using two-tailed Student's *t* test.

Supplementary Table 7. Patient information of spleen donors used in this study.

	Young (n=10)	Old (n=10)	p value
**Sex: Male (%)**	9 (90.0)	8 (80.0)	0.53[a]
**Age: Median [range]**	29 [20-36]	73 [70-87]	< 0.0001[b]
**Diagnosis (%)**			0.020[a]
**Pseudocyst**	1 (10.0)	0 (0.0)	
**Trauma**	8 (80.0)	3 (30.0)	
**Tumor**	1 (10.0)	7 (70.0)	

[a]*p* values are obtained using Chi-square test. [b]*p* value is obtained using two-tailed Student's *t* test.

Supplementary Figure Legends

Supplementary Fig. 1. The distribution of senescent cells in the normal tissues from young and old individuals. a The quantification data for the proportion of $p16^{INK4A+}$ cells in the tissue parenchyma and stroma. **b** The mRNA expression level of *IFNA1* and *IFNB1* in young ($p53^{-}/p21^{Waf1-}/p16^{INK4A-}$), intermediate stage of senescence ($p53^{+}/p21^{Waf1+}/p16^{INK4A-}$), and fully senescent ($p53^{+}/p21^{Waf1+}/p16^{INK4A+}$) *in vitro*. **c** IHC analysis of $p16^{INK4A}$ and IFNα in colonic stroma from the elderly individuals. The red arrows indicate $p16^{INK4A-}$ cells and the black arrow indicate $p16^{INK4A+}$ cells, respectively. **d** IHC analysis of $p16^{INK4A}$ and IFNβ in colonic stroma from the elderly individuals. The red arrows indicate $p16^{INK4A-}$ cells and the black arrow indicate $p16^{INK4A+}$ cells, respectively. **e** The IHC analysis for H3K9me3 in colon and lung from young and old individuals, respectively (upper panel). The lower panel shows the corresponding quantification data. **f** Double IF staining for $p16^{INK4A}$ and $p21^{Waf1}$. A white and yellow arrows indicate $p16^{INK4A+}$and $p21^{Waf1+}$ cells, respectively. All graphs are shown as mean ± standard deviation. The *p* value is obtained using Mann-Whitney U test.

Supplementary Fig. 2. The distribution of $Ki67^{+}$ cells in normal tissues from young and old individuals. The quantification data for the proportion of $Ki67^{+}$ cells in the tissue parenchyma and stroma. All graphs are shown as mean ± standard deviation. The *p* value is obtained using Mann-Whitney U test.

Supplementary Fig. 3. The multiplex IHC for $p16^{INK4A}$ and Ki67 in normal colon from young and old individuals. The multiplex IHC analysis for $p16^{INK4A}$ (red) and Ki67 (brown) in normal colon tissues from young and old individuals are shown, respectively.

Supplementary Fig. 4. The distribution of senescent cells in normal tissues from young and old mouse. The quantification data for the proportion of $p16^{INK4A+}$ cells in the tissue parenchyma and stroma. All graphs are shown as mean ± standard deviation.

Supplementary Fig. 5. scRNAseq results of GSE178341 (colon) and GSE130973 (skin). a Dimension plots of GSE178341 according to the Seurat cluster (left panel) and the patient number (right panel) are shown. **b** The feature plots for representative cell type markers of GSE178341 are shown. **c** Dimension plots of GSE130973 according to the Seurat cluster (left panel) and the patient age (right panel) are shown. **d** The expression of cell type markers in GSE130973 is shown using violin plots.

Supplementary Fig. 6. scRNAseq results of SYN21041850 (lung) and GSE149614 (liver). a Dimension plots of GSE178341 according to the Seurat cluster (left panel) and the patient number (right

panel) are shown. **b** The plots for representative cell type markers of SYN21041850 are shown using violin plots. **c** Dimension plots of GSE149614 according to the Seurat cluster (left panel) and the patient age (right panel) are shown. **d** The expression of cell type markers in GSE149614 is shown using violin plots.

Supplementary Fig. 7. scRNAseq results of GSE109774 (Tabula Muris) a A dimension plot shows cells from various organs of young (3-month-old) and aged mouse (18-month and 24-month-old) (left panel). A feature plot shows the expression of *Cdkn2a* in whole mouse cells (right panel). **b** Dot plots shows the expression levels of *Cdkn2a* in various mouse organs according to age of mouse.

Supplementary Fig. 8. scRNAseq results of lung and colon tissues in GSE109774 (Tabula Muris) a A dimension plot shows the whole cells from various mouse tissues (left panel). A dimension plot shows the fibroblasts from various mouse tissues (middle panel). The violin plots show the *Cdkn2a* expression in fibroblasts according to age of mouse (right panel). **b** A dimension plot shows the whole cells from the lung tissue according to age of mouse (left and middle panel). The violin plots show the *Cdkn2a* expression in various cell types in lung according to age of mouse (right panel). **c** A dimension plot shows the whole cells from the colon tissue according to age of mouse (left and middle panel). The violin plots show the *Cdkn2a* expression in various cell types in colon according to age of mouse (right panel).

Supplementary Fig. 9. The *Cdkn2a* expression level in skin, lung, liver, and colon tissues in GSE109774 (Tabula Muris) a-d Dimension plots show various cell types in mouse tissues, respectively (left panel). Feature plots show the expression of *Cdkn2a* in mouse tissues, respectively (right panel).

Supplementary Fig. 10. The cell-to-cell interaction networks of normal colon tissue in GSE178341. The interaction strength of MIF (left panel) and IL16 (right panel) signaling pathways in GSE178341.

Supplementary Fig. 11. The proteases expression level in young and senescent T cells. The mRNA expression level of various proteases (Cathepsin G, Thrombin, proteinase 3, plasmin, MMP1 and MMP2), known to cleave PARs, were examined by real-time PCR in young and senescent T cells (sen). ND: not detected. The graph is shown as mean ± standard deviation. The p value is obtained using Mann-Whitney U test.

Supplementary Fig. 12. Caspase 3/7 activation mediated to GzmA-PAR1 axis in human colon epithelial cells. Human colon epithelial cells were pre-treated with PAR1 antagonist for 1h and

incubated with recombinant human GzmA (rhGzmA) protein for 1 day. Caspase 3/7 activity was measured by luminescence-based assay. The graph is shown as mean + standard deviation. The *p* value is obtained using Mann-Whitney U test.

Supplementary Fig. 13. Nafamostat mesylate (NM) treatment with CM from senescent T cells or rhGzmA decreases PAR1 and PAR2 signaling pathway. NM was incubated with CM from senescent T cells (sen CM) or recombinant human granzyme A protein (50 ng/ml) at indicated concentration for 1h. These mixtures were treated to human colonic epithelial cells for 1 day. Caspase 3/7 activity and IL8 mRNA level were measured by luminescence-based assay and real-time PCR, respectively. The graph is shown as mean + standard deviation. The p value is obtained using Mann-Whitney U test.

Supplementary Fig. 14. The IL8 expression associated with GzmA-PAR2 axis in human colon epithelial cells. Human colon epithelial cells were pre-treated with PAR2 antagonist for 1h and then incubated with recombinant human GzmA protein for 1 day. The IL8 mRNA expression was analyzed by real-time PCR. The graph is shown as mean + standard deviation. The *p* value is obtained using Mann-Whitney U test.

Supplementary Fig. 15. The pro-inflammatory cytokine expression in GzmA-treated human colon epithelial cells. Human colon epithelial cells were treated with recombinant human GzmA protein for 1 day. Pro-inflammatory cytokines/chemokines (IL6, IL1β, CCL2 and TNF-α) expression were analyzed by real-time PCR. The graph is shown as mean + standard deviation. The *p* value is obtained using Mann-Whitney U test.

Supplementary Fig. 16. GzmB expression in young and senescent T cells. The GzmB mRNA expression was detected by real-time PCR in young and senescent T cells. The graph is shown as mean + standard deviation. The *p* value is obtained using Mann-Whitney U test. ‘Young’ and ‘Sen’ indicate the young and senescent T cells, respectively.

Supplementary Fig. 17. $p16^{INK4A}$ IHC analysis in normal spleen from young and elderly tissues. IHC analysis for $p16^{INK4A}$ in normal spleen germinal center from young and elderly individuals (left panel). The middle panel displays the number quantification data of $p16^{INK4A+}$ cells. The multiplex IHC analysis for $p16^{INK4A}$ and CD3 in human aged spleen germinal center (right panel). The graph is shown as mean ± standard deviation. The *p* value is obtained using Mann-Whitney U test. ‘Young’ and ‘Old’ indicate the young and the elderly individuals, respectively.
